# *GRP-3* and *KAPP,* encoding interactors of WAK1, negatively affect defense responses induced by oligogalacturonides and local response to wounding

**DOI:** 10.1093/jxb/erv563

**Published:** 2016-01-08

**Authors:** Giovanna Gramegna, Vanessa Modesti, Daniel V. Savatin, Francesca Sicilia, Felice Cervone, Giulia De Lorenzo

**Affiliations:** Istituto Pasteur-Cenci Bolognetti, Dipartimento di Biologia e Biotecnologie ‘C. Darwin’, Sapienza Università di Roma, Piazzale Aldo Moro 5, 00185 Rome, Italy

**Keywords:** *Arabidopsis thaliana*, GRP-3, KAPP, oligogalacturonides, oxidative burst, pathogen resistance, Wall-associated kinase, wounding.

## Abstract

Conserved microbe-associated molecular patterns (MAMPs) and damage-associated molecular patterns (DAMPs) act as danger signals to activate the plant immune response. These molecules are recognized by surface receptors that are referred to as pattern recognition receptors. Oligogalacturonides (OGs), DAMPs released from the plant cell wall homogalacturonan, have also been proposed to act as local signals in the response to wounding. The Arabidopsis Wall-Associated Kinase 1 (WAK1), a receptor of OGs, has been described to form a complex with a cytoplasmic plasma membrane-localized kinase-associated protein phosphatase (KAPP) and a glycine-rich protein (GRP-3) that we find localized mainly in the cell wall and, in a small part, on the plasma membrane. By using Arabidopsis plants overexpressing WAK1, and both *grp-3* and *kapp* null insertional mutant and overexpressing plants, we demonstrate a positive function of WAK1 and a negative function of GRP-3 and KAPP in the OG-triggered expression of defence genes and the production of an oxidative burst. The three proteins also affect the local response to wounding and the basal resistance against the necrotrophic pathogen *Botrytis cinerea*. *GRP-3* and *KAPP* are likely to function in the phasing out of the plant immune response.

## Highlight

*WAK1* and its interactors *GRP-3* and *KAPP* play a role in elicitor-induced gene expression, local response to wounding, and pathogen resistance.

## Introduction

An efficient sensing of danger and a rapid activation of the immune response are crucial for the survival of plants. Plants defend themselves against pathogenic micro-organisms by detecting conserved microbial molecules that are referred to as microbe- or pathogen-associated molecular patterns (MAMPs or PAMPs). Perception of PAMPs by pattern recognition receptors (PRRs) localized on the plasma membrane activates downstream events leading to resistance (Boller and Felix, 2009; Zipfel, 2014). These include ion fluxes, the production of reactive oxygen species (ROS), the activation of mitogen-activated and calcium-dependent protein kinases, the induction of defence gene expression, and callose deposition (Zipfel *et al.*, 2006; Colcombet and Hirt, 2008; Nicaise *et al.*, 2009; Boudsocq *et al.*, 2010; Luna *et al.*, 2011). The activated responses culminate in the so-called PAMP-triggered immunity (PTI), which confers resistance to a broad range of pathogens (Jones and Dangl, 2006; Boller and Felix, 2009). In *Arabidopsis thaliana*, the best-studied PRRs are the leucine-rich repeat receptor kinases (LRR-RKs) FLAGELLIN SENSING2 (FLS2) and EF-Tu receptor (EFR) that specifically bind the bacterial peptides flg22 (derived from flagellin) and elf18 (derived from the elongation factor Tu), respectively (Chinchilla *et al.*, 2006; Zipfel *et al.*, 2006).

In addition to PAMPs, PRRs also perceive signals referred to as damage-associated molecular patterns (DAMPs) (Boller and Felix, 2009; De Lorenzo *et al.*, 2011; Heil and Land, 2014). These are plant endogenous molecules that are released upon cell damage caused by pathogens or mechanical stress. A well-characterized class of DAMPs is represented by oligogalacturonides (OGs), linear oligomers of α-1,4 d-galacturonic acid residues with a degree of polymerization (DP) ranging from 10 to 16, released from non-methylated homogalacturonan, i.e. the main component of the plant cell wall pectin. OGs can be released early during infection by pectin degrading enzymes, especially *endo*-polygalacturonases (PGs), which are secreted by pathogenic microbes (Ridley *et al.*, 2001; De Lorenzo *et al.*, 2011; Ferrari *et al.*, 2013; Benedetti *et al.*, 2015), and their accumulation is favoured, both *in vitro* and *in vivo*, by the interaction with specific inhibitors of PGs, named polygalacturonase-inhibiting proteins (PGIPs) (Casasoli *et al.*, 2009; Cervone *et al.*, 2015; Kalunke *et al.*, 2015).

Treatment with OGs triggers plant responses that overlap those induced by PAMPs, i.e. an oxidative burst (Galletti *et al.*, 2008), the activation of MAPKs (Galletti *et al.*, 2011; Savatin *et al.*, 2014*a*), the induction of glucanase and chitinases (Davis and Hahlbrock, 1987; Broekaert and Pneumas, 1988) and a wide reprogramming of gene expression (Denoux *et al.*, 2008) including the inhibition of auxin-regulated responses (Bellincampi *et al.*, 1996; Savatin *et al.*, 2011). It also confers protection against the necrotrophic fungus *Botrytis cinerea* in grapevine (*Vitis vinifera*) and Arabidopsis (Aziz *et al.*, 2004; Ferrari *et al.*, 2007; Suarez *et al.*, 2013; Cervone *et al.*, 2015; Gravino *et al.*, 2015), indicating that the action of these elicitors at the site of infection is important for plant immunity. Recently, it has been demonstrated that OGs released *in vivo* act as a DAMP signal to trigger plant immunity. Indeed, transgenic plants expressing, in a pathogen-inducible manner, a protein fusion, between a fungal PG and a plant PGIP (named OG machine, OGM), and capable of enhancing the levels of OGs in the tissues, are more resistant to *B. cinerea*, *Pectobacterium carotovorum*, and *Pseudomonas syringae* (Benedetti *et al.*, 2015; Cervone *et al.*, 2015).

OGs have been proposed as wounding signals since they induce the accumulation of a proteinase inhibitor in tomato where a wound-inducible PG gene may be responsible for their production (Ryan and Jagendorf, 1995; Bergey *et al.*, 1999; Heil and Land, 2014; Savatin *et al.*, 2014*b*). Because OGs, due to their anionic nature, have a very limited mobility in the plant apoplast, their activity as a wound signal is likely to be restricted to areas that are close to the wounded tissue (Baydoun and Fry, 1985). The Arabidopsis Wall-associated Kinase 1 (WAK1) has been identified as an OG receptor (Brutus *et al.*, 2010). WAK1 is a receptor like kinase (RLK) that belongs to a family of five members (WAK1–5), whose encoding genes are tightly clustered on the chromosome 1 (He *et al.*, 1999). The role of *WAK1* in immunity is difficult to prove by using insertional or silenced lines due to functional redundancy. In particular, Arabidopsis knock-out mutants for individual *WAK* genes do not show significant alterations, and the generation of double or multiple mutants is difficult because the genes are tightly clustered (He *et al.*, 1999). Moreover, transgenic plants constitutively expressing *WAK1* or *WAK2* antisense transcripts, which silence the whole family, could not be obtained, suggesting that loss of the WAK function determines lethality (Wagner and Kohorn, 2001). Plants expressing an inducible full-length antisense *WAK*, which leads to a reduction of total WAK protein levels, show a loss of cell expansion, whereas plants with inducible silencing of individual *WAK1* and *WAK2*, using gene-specific antisense transcripts show no observable phenotypic alterations, suggesting functional redundancy (Wagner and Kohorn, 2001). Pathogen resistance of these lines has never been assessed.

Interestingly, *WAK1* is the only member of the family that is up-regulated in response to OGs (Denoux *et al.*, 2008); moreover, *WAK1* is also induced by wounding (Wagner and Kohorn, 2001) and transgenic plants overexpressing WAK1 are more resistant to *B. cinerea* (Brutus *et al.*, 2010). The extracellular domain of WAK1 has been shown to interact with the putatively apoplastic glycine-rich protein GRP-3, and the GRP-3/WAK1 complex interacts with the cytosolic kinase associated protein phosphatase KAPP, as demonstrated by gel filtration and co-immunoprecipitation analyses (Anderson *et al.*, 2001; Park *et al.*, 2001). Very little is known about the biological role of GRP-3 in plants (Fusaro *et al.*, 2001; Bocca *et al.*, 2005; Mangeon *et al.*, 2009). Arabidopsis GRP-3 shows similarity to the soybean noduline-24 and belongs to class II of the GRPs, characterized by a GGxxxGG motif and a cysteine-rich C-terminal domain necessary for its interaction with WAK1 (De Oliveira *et al.*, 1990; Park *et al.*, 2001). The signal peptide for translocation of GRP-3 into the endoplasmic reticulum (ER) also shows similarity to that of noduline-24 and suggests an apoplastic localization of the protein. Transcripts of *GRP-3* are very abundant in stems and leaves (De Oliveira *et al.*, 1990), where transcripts of *WAK1* are also present (He *et al.*, 1999; Wagner and Kohorn, 2001). KAPP belongs to the Mg^2+^/Mn^2+^-dependent protein phosphatase family and carries an N-terminal type I signal anchor followed by a kinase interaction (KI) domain and a C-terminal type 2C-protein phosphatase catalytic domain (Stone *et al.*, 1994). The KI domain binds *in vitro* the kinase domain of different RLKs in a phosphorylation-dependent manner and does not bind kinase-inactive mutants of RLKs (Williams *et al.*, 1997; Stone *et al.*, 1998). In addition to WAK1, KAPP interacts with several RLKs, such as CLAVATA1 (Williams *et al.*, 1997; Trotochaud *et al.*, 1999) and SOMATIC EMBRYOGENESIS RECEPTOR-LIKE KINASE1 (Shah *et al.*, 2002) as well as FLS2, at the level of the kinase domain, negatively regulating flagellin signalling (Gomez-Gomez *et al.*, 2001). The functional characterization of a loss-of-function mutant of KAPP (*rag1*, root attenuated growth1) also suggested that KAPP is a component of a novel Na^+^ adaptation pathway (Manabe *et al.*, 2008).

In this work, we show that individual loss of *GRP-3* and *KAPP* leads to a prolonged gene expression induced by OGs and flg22, enhanced local response to wounding, and basal resistance against fungal necrotrophic pathogens. The same phenotype was observed in plant overexpressing WAK1, pointing to a negative role of GRP-3 and KAPP on elicitor-induced immunity. On the other hand, individual overexpression of the two WAK1 interactors confirms the negative role of KAPP on both OG and flg22 signalling and reveals the potential of GRP-3 to enhance responsiveness to OGs and repress that to flg22.

## Materials and methods

### Plant materials

Wild-type seeds of *Arabidopsis thaliana* ecotype Columbia-0 (Col-0) were purchased from Lehle Seeds. Col-0 *efr* seeds were kindly provided by Dr Zipfel (The Sainsbury Laboratory, Norwich, UK). Seeds of *kapp* (SAIL_1255-D05), *kapp-2* (SALK_126141.54.75), and *grp-3* (SALK_084685.46.60) insertional mutants were purchased from the European Arabidopsis Stock Centre. Homozygous mutants were isolated by PCR-based genotyping using the gene-specific PCR primers listed in Supplementary Table S1 at *JXB* online.

### Generation of transgenic plants

*WAK1* and *GRP-3* full-length cDNA clones were obtained from the Riken BioResource Center. *WAK1* and *GRP3* were cloned in-frame with and upstream of the EGFP or RFP coding sequence. The Multisite Gateway Recombination Cloning Technology (Life Technologies) was used to generate WAK1-EGFP. In particular, a pEN-WAK1 entry clone was generated in the pDONR221/Zeo vector (Life Technologies). Multisite recombination was then performed by using the pEN-L4-2-R1 and the pEN-R2-F-L3 vectors, which contain the 35S promoter and the EGFP coding sequence, respectively, and pB7m34GW as the destination binary vector which confers phosphinothricin resistance. To generate the 35S::GRP3-RFP construct, the cDNA sequence encoding GRP-3 was amplified by PCR and cloned into a *Hin*dIII- and *Bam*HI- cleaved pSAT6-RFP-N1 vector resulting in GRP3-RFP construct. The plasmid was verified by restriction endonuclease digestion and DNA sequencing and the GRP3-RFP sequence was amplified and cloned in the pH2GW7 binary vector, which confers hygromycin resistance, using the Gateway Recombination Cloning Technology. All Gateway compatible vectors were previously described (Karimi *et al.*, 2002) and obtained from Plant Systems Biology (Ghent University; http://gateway.psb.ugent.be/). The *EFR* full-length coding sequence was amplified by PCR from genomic DNA extracted from 10-d-old Col-0 seedlings and introduced into the *Sma*I and *Pac*I restriction sites of the pBI121 vector, which confers kanamycin resistance. Primer sequences used to generate all the constructs are shown in Supplementary Table S1. Constructs were verified by sequencing (PRIMM; Milano, Italy). The construct 35S:KAPP–YFP was kindly provided by Professor Elliot M Meyerowitz (California Institute of Technology, Pasadena).

Stable transgenic lines were obtained using the standard *Agrobacterium tumefaciens*-mediated gene transfer procedure (floral dip) (Clough and Bent, 1998), using the *A. tumefaciens* GV3101 strain. The independent transgenic lines obtained were selected based on their antibiotic resistance. For all lines, homozygous plants of the T_3_ generation, carrying a single transgene insertion, were obtained for analysis.

### Growth conditions and treatments

Arabidopsis plants were grown in soil (Compo Sana) at 22 °C and 70% relative humidity under a 12/12h light/dark cycle (approximately 120 μmol m^−2^ s^−1^). For elicitor treatments in adult plants, 4-week-old plants were sprayed with H_2_O, OGs (50 μg ml^−1^), elf18 (100nM), flg22 (100nM), and OG3 (50 μg ml^−1^). For seedling assays, seeds were surface sterilized and germinated in multi-well plates (approximately 10 seeds well^–1^) containing 0.5× Murashige and Skoog (MS; Murashige and Skoog, 1962) medium supplemented with 0.5% sucrose (2ml well^–1^). For gene expression analysis, seedlings were grown at 22 °C and 70% relative humidity under a 16/8h light/dark cycle (approximately 120 μmol m^−2^ s^−1^). After 9 d, the medium was adjusted to 1ml and treatments with OGs (10 and 50 μg ml^−1^, final concentrations) and flg22 (10nM) were performed after 24h. Wounding was performed on leaves from 4-week-old plants by applying a single pressure on the middle of the leaf lamina with a laboratory forceps. At least three wounded leaves from four different plants for each biological replicate were used for wound-induced callose deposition analysis. Analysis of the expression of wound-responsive genes was performed on two leaves from three different plants, 30min and 60min after wounding.

### Gene expression analysis

Gene expression analyses were performed as previously described by Savatin *et al.* (2014*a*). Primer sequences are shown in Supplementary Table S2 at *JXB* online.

### Callose deposition

The analysis of callose deposition was performed as previously described with slight modifications (Brutus *et al.*, 2010). Callose deposition in spraying and wounding experiments was evaluated using two different scoring systems, due to the different deposition patterns observed in response to the two treatments. After elicitor spraying, callose was deposited on the whole leaf lamina with differences regarding the density, distribution, and the shape of the deposits, and the scoring scale considered all these features. In the wounding experiments, a different scoring scale was used because of the different callose deposition pattern, i.e. callose was present only at the edge of the wounded tissue in the wild-type plants and was also observed in the area surrounding the wound site, but only up to a distance of 0.5–1mm (proximal region), in the mutant/transgenic plants.

### Measurement of hydrogen peroxide

Hydrogen peroxide generated by seedlings in response to OGs and flg22 (50 μg ml^−1^ and 100nM, respectively) was measured in the incubation medium by a colorimetric assay based on the xylenol orange dye (*o*-cresolsulphonephthalein 3′,3″-bis[methylimino] diacetic acid, sodium salt; Sigma), as previously described by Galletti *et al.* (2008). Fourteen-day-old seedlings were used to have more plant biomass and to increase the level of hydrogen peroxide produced, for a more reproducible detection. Hydrogen peroxide produced by leaf discs was measured by a luminol-based assay as previously described by Savatin *et al.* (2014*a*).

### Pathogen infections

Pathogen infections were conducted on rosette leaves of 4-week-old plants. *B. cinerea* growth and inoculation was performed as previously described by Savatin *et al.* (2014*a*). For OG-induced protection experiments, plants were sprayed with water or 200 μg ml^–1^ OGs 24h before inoculation, as previously described by Savatin *et al.* (2014*a*). *Pectobacterium carotovorum* subsp. *carotovorum* (strain DSMZ 30169) was obtained from DSMZ GmbH (Braunschweig, Germany). Bacteria were cultivated in Luria–Bertani (LB) liquid medium (Duchefa Biochemie, Haarlem, The Netherlands) for 16–18h at 28 °C, 340rpm. Bacteria were then collected by centrifugation (8 000×*g* for 10min), washed twice in 50mM potassium-phosphate buffer (pH 7.0) and suspended at the desired concentration (for example, a final OD_600_=0.001 corresponded to a concentration of 5×10^6^ colony forming units ml^−1^). Infections were performed on intact Arabidopsis plants: leaves were punctured with a sterile needle on the epidermis of the adaxial surface of each leaf, at the sides of the mid-rib in the central part of the leaf. A 5 µl droplet of the bacterial suspension was placed on each punctured site. Plants were kept at 22 °C and 70% relative humidity under a 12/12h light/dark cycle (approximately 120 μmol m^−2^ s^−1^). The area of water-soaked lesions was determined 24h after inoculation. Analyses were performed in at least two independent biological replicates, each comprising at least 24 lesions (three leaves per plant and at least four plants per genotype).

### Laser scanning and spinning disc confocal microscopy analyses

Seedlings were grown for 6 d in Petri dishes containing MS medium agar plates supplemented with 1% sucrose and detached cotyledons were analysed. Localization analysis was performed using an inverted laser scanning confocal microscope (LSM780 NLO; Carl Zeiss). For co-localization experiments, detached cotyledons were stained for 15min with 4mM FM4-64 (Life Technologies) (Brandizzi *et al.*, 2004). The Zeiss ZEN confocal software was used for post-acquisition image processing. An inverted spinning-disc confocal microscope (CarvX, CrEST) was used for the plasmolysis experiments of the GRP-3-RFP seedlings. Imaging was performed using a CFI Planfluo 40× (1.4 Numerical Aperture) oil immersion objective (NIKON) through a 70 µm pinhole disc set at 6 000rpm. Detection was performed using a cooled charge-coupled device CCD camera (CoolSNAP HQ2, Photometrics) and omega band-pass filters XF101-2 (for RFP). The CCD camera, Z-motor, and Confocal head were controlled by Metamorph software (Molecular Devices).

## Results

### Duration of elicitor-induced gene expression is increased in *grp-3* and *kapp* loss-of function mutants

In order to elucidate the role of *KAPP* and *GRP-3* in OG signalling, responses typically induced by OGs were analysed in single mutant lines carrying T-DNA insertions in the corresponding genes. Two allelic insertional mutants (*kapp* and *kapp-2*) and a single *grp-3* mutant, all in the Col-0 background, were characterized (see Supplementary Fig. S1A, B at *JXB* online). A single insertion was present in each mutant, as shown by segregation analysis of the antibiotic resistance gene in the progeny of heterozygous plants (3:1, resistant:susceptible) from which homozygous lines were obtained. Seedlings of *kapp* and *grp-3* mutants showed no expression of full length transcripts of the corresponding genes and therefore represent null mutants (Supplementary Fig. S1C); *kapp-2* instead showed a reduced level of *KAPP* transcripts (about 14% compared with the wild type) and therefore represents a knock-down mutant (Supplementary Fig. S1D).

In parallel, because loss-of-function *WAK1* mutants do not show significant alterations of immune responses, response to elicitors was examined in homozygous transgenic Col-0 Arabidopsis plants expressing WAK1 fused to the Enhanced Green Fluorescent Protein (EGFP) under the control of the CaMV 35S promoter. Among four independent transgenic lines that showed 3:1 segregation of the antibiotic resistance in the T_2_ progeny and the highest levels of the transgene transcripts (see Supplementary Fig. S2A at *JXB* online), lines 2 and 4 were chosen for further analysis; the latter line is indicated simply as WAK1 lines or plants. Gene expression analysis by quantitative RT-PCR (qRT-PCR) confirmed that *WAK1,* evaluated as the additive contribution of endogenous plus transgene transcripts, is overexpressed in seedlings and adult leaves of both WAK1 lines, compared with the wild type, with higher levels observed in leaves (Supplementary Fig. S2B). All mutants and transgenic plants showed no obvious morphological and developmental phenotypes.

**Fig. 1.**
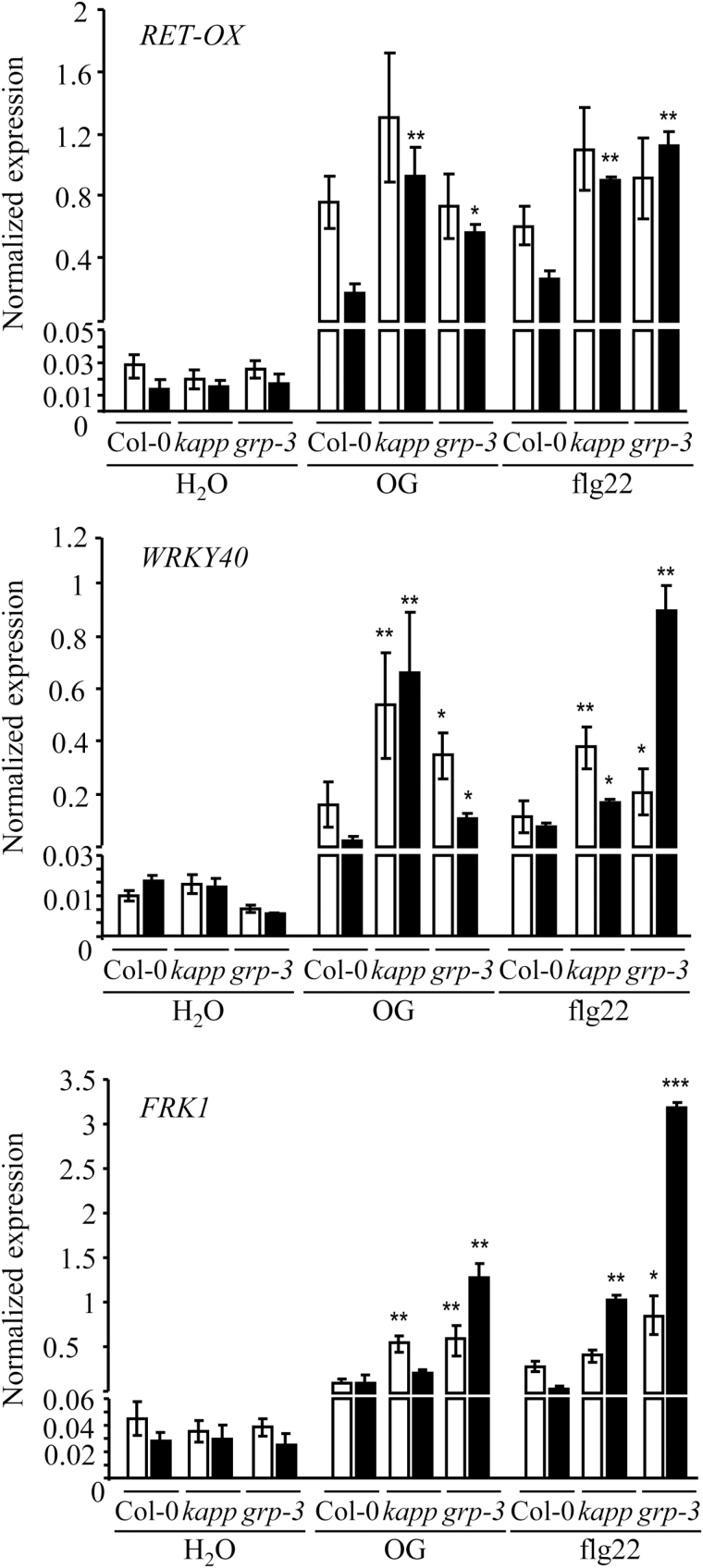
Seedlings of *kapp* and *grp-3* mutants show a prolonged expression of defence response genes in response to elicitors. Seedlings were treated with OGs (50 μg ml^−1^) or flg22 (10nM) or water, as a control, and accumulation of *RET-OX*, *WRKY40*, and *FRK1* transcripts was analysed after 1h (white bars) and 3h (black bars) by quantitative RT-PCR, using *UBQ5* for normalization. Transcript levels are expressed as the gene/*UBQ5* ratio (normalized expression). Values are means (±SE) of three independent experiments (*n*=20, in each experiment). Asterisks indicate statistically significant differences between elicitor treatment of mutant seedlings and Col-0, according to Student’s *t* test (*, *P* <0.05; **, *P* <1×10^−3^; ***, *P* <5×10^−4^).

The expression of genes that are markers of the elicitor response (Denoux *et al.*, 2008; Galletti *et al.*, 2008) was monitored in *kapp*, *kapp-2*, *grp-3*, WAK1, and wild-type seedlings upon treatment with OGs, flg22 as a representative MAMP, or water as a control for 1h and 3h. The genes analysed were *RetOx*, encoding a protein with homology to reticuline oxidases (Denoux *et al.*, 2008; Galletti *et al.*, 2008), *WRKY40*, encoding a transcription factor that acts as a negative regulator of basal defence responses, and *FRK1*, encoding a flg22-induced receptor-like kinase. Upon treatment of seedlings with OGs or flg22, the expression of *RetOx* is known to peak at 1h and to decrease at 3h, whereas the expression of *WRKY40* and *FRK1* peaks at 30min and decreases nearly to basal levels at 1h (Denoux *et al.*, 2008; Galletti *et al.*, 2008). In the water-treated *kapp* and *grp-3* mutant seedlings, levels of all marker gene transcripts were comparable with those of the wild type. Upon elicitation with OGs or flg22, accumulation of *RetOx* transcripts at 3h in both mutants was higher than in the wild type (Fig. 1), and accumulation of *WRKY40* transcripts was higher at both 1h and 3h. A higher OG- and flg22-induced expression of both *RetOx* and *WRKY40* at 3h was also observed in *kapp-2* seedlings (see Supplementary Fig. S3A at *JXB* online). The expression of *FRK1* was higher than in Col-0 at 1h after treatment with OGs and at 3h after treatment with flg22 in the *kapp* mutant, and at both time points in response to the two elicitors in *grp-3* (Fig. 1). These data show a more prolonged expression of the marker genes upon elicitation with OGs and flg22 in the two types of mutants and suggest a negative role of *KAPP* and *GRP-3* in elicitor-induced immunity. In WAK1 seedlings, however, the elicitor-induced expression of *RetOx, WRKY40*, and *FRK1* transcripts was similar to that of the wild type at two different concentrations of OGs (10 and 50 μg ml^−1^) (see Supplementary Fig. S4 at *JXB* online).

### Elicitor-induced production of extracellular hydrogen peroxide is increased in *kapp*, *grp-3*, and WAK1 plants

**Fig. 2.**
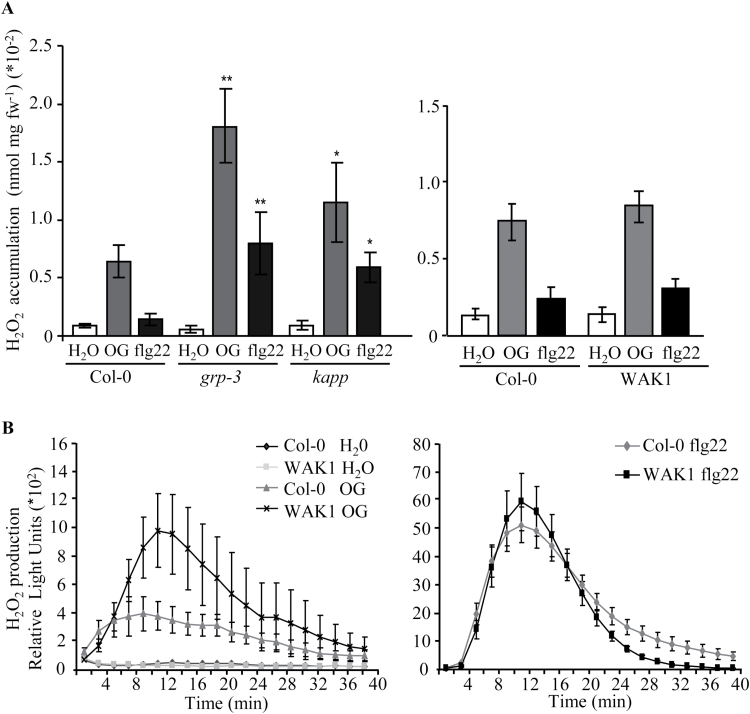
Elicitor-induced production of extracellular hydrogen peroxide in *grp-3*, *kapp* and WAK1 plants. (A) Seedlings of *grp-3*, *kapp*, and WAK1 (#4) lines were treated with water (white bars), OGs (50 μg ml^−1^, grey bars), and flg22 (100nM, black bars) and the accumulation of hydrogen peroxide in the culture medium was measured by a xylenol orange-based assay. Results are means of four independent experiments (±SE; *n*=40 in each experiment). Asterisks indicate statistically significant difference between control and mutant/transgenic plants, according to Student’s *t* test (*, *P* <5×10^−4^; **, *P* <5×10^−6^). fw, fresh weight. (B) hydrogen peroxide production was measured using a luminol-based assay in leaf discs from WAK1 (line #4) and Col-0 plants after treatment with water and OGs (150 μg ml^−1^; left) or flg22 (10 μM; right). Results are mean ±SE of three independent experiments (*n*=12). Results obtained with the WAK1 #2 transgenic line are shown in Supplementary Fig. S5B.

Because the production of extracellular ROS is one of the first measurable responses to elicitors (Bailey-Serres and Mittler, 2006; Galletti *et al.*, 2008), accumulation of H_2_O_2_ was analysed in *kapp, kapp-2*, *grp-3*, and WAK1 seedlings treated with OGs, flg22, and water using a xylenol orange-based colorimetric assay. In agreement with previous data, our experiments showed that H_2_O_2_ produced in response to flg22 is lower than that produced in response to OGs (Bailey-Serres and Mittler, 2006; Galletti *et al.*, 2008). After treatment with both elicitors, the level of H_2_O_2_ produced by *kapp, kapp-2*, and *grp-3* seedlings was significantly higher than that produced by wild-type seedlings (Fig. 2A; Supplementary Fig. S3B). No difference was observed in H_2_O_2_ produced by WAK1 seedlings in response to both elicitors (Fig. 2A; see Supplementary Fig. S5A at *JXB* online).

A low expression of the WAK1 transgene in the seedlings may account for the comparable response to OGs of WAK1 and wild-type seedlings. Because expression of the transgene is higher in leaves (Supplementary Fig. S2B), we decided to examine the OG-induced oxidative burst of WAK1 plants in leaf discs using a luminol/peroxidase-based assay. Accumulation of H_2_O_2_ was significantly higher in the WAK1 leaves than in the wild-type leaves after treatment with OGs, but not with flg22 or water (Fig. 2B; Supplementary Fig. S5B).

### Callose deposition in response to sprayed elicitors is increased in *kapp*, *grp-3*, and WAK1 plants

**Fig. 3.**
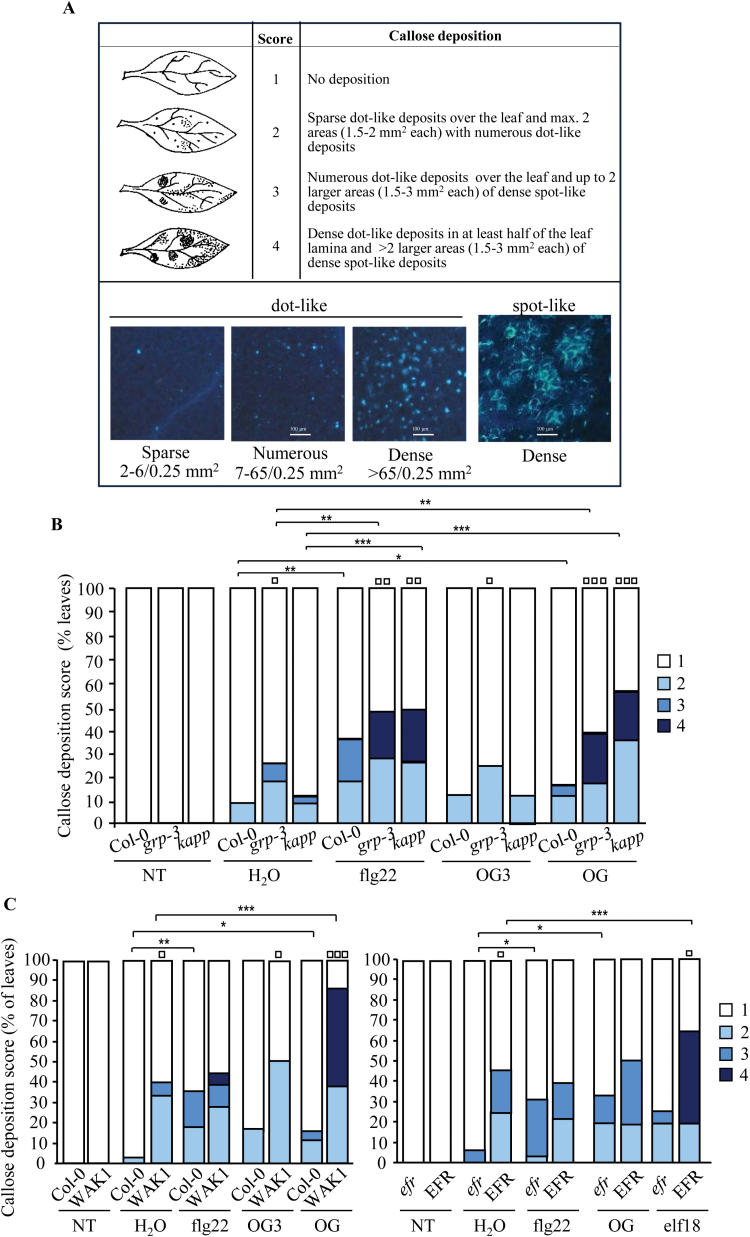
Callose deposition in Arabidopsis *grp-3*, *kapp*, WAK1 and EFR plants in response to sprayed MAMPs and DAMPs. Plants were sprayed with water, flg22 (100nM), short and biologically inactive OGs (OG3, 50 μg ml^−1^), and OG (50 μg ml^−1^). Callose deposition was examined in leaves and is expressed as a score, as indicated in (A). Bars, 100 μm. The histograms in (B) and (C) show the percentage of leaves with a specific callose deposition score. White squares directly above the bars indicate statistically significant difference between mutant/transgenic plants and control plants (Col-0 for *kapp*, *grp-3*, and WAK1 plants, and Col-0 *efr* for EFR plants). Asterisks above the connection lines indicate statistically significant difference between water and elicitors treatment in each genetic background. Statistical analysis was performed according to Fisher’s exact test (* or white square *P* <0.05; **, *P* <5×10^−3^; *** *P* <1×10^−4^). Five independent experiments (*n*=12 in each experiments) were performed.

Deposition of callose, i.e. one of the late defence responses activated by both MAMPs and DAMPs (Galletti *et al.*, 2008; Luna *et al.*, 2011), was examined in leaves of *kapp*, *grp-3*, and WAK1 plants. In parallel, the same response was examined in leaves of Col-0 plants transformed with the empty vector (EV plants) as well as in plants expressing a CaMV 35S::EFR gene construct in the Col-0 *efr* background (EFR plants), both used as controls. The level of *EFR* transcripts in leaves of EFR plants was about 7-fold higher than in Col-0 leaves (Supplementary Fig. S2C). Plants were sprayed with water, OGs, flg22 or a commercial trimer (OG3) that is inactive as an elicitor in our assays, and callose deposition in leaves was examined after 24h by aniline blue staining (Fig. 3A). Treatment with OGs and flg22 induced a very weak or, in most cases, no response in wild-type plants, and induced a more intense callose deposition in both the *kapp* and *grp3* mutants; elicitor-inactive OGs did not induce a response significantly different from that of water (Fig. 3B). A very intense callose deposition was observed in WAK1 plants only in response to OGs while the response to flg22 was comparable to that of wild-type plants (Fig. 3C; Supplementary Fig. S5C). Control EFR plants showed a wild-type-like response to sprayed OGs or flg22 and an increased response to elf18, the ligand of EFR (Fig. 3C). Control EV plants showed a response similar to that of wild-type plants (see Supplementary Fig. S6A at *JXB* online).

### *WAK1, GRP-3*, and *KAPP* play a role in the local response to wounding

**Fig. 4.**
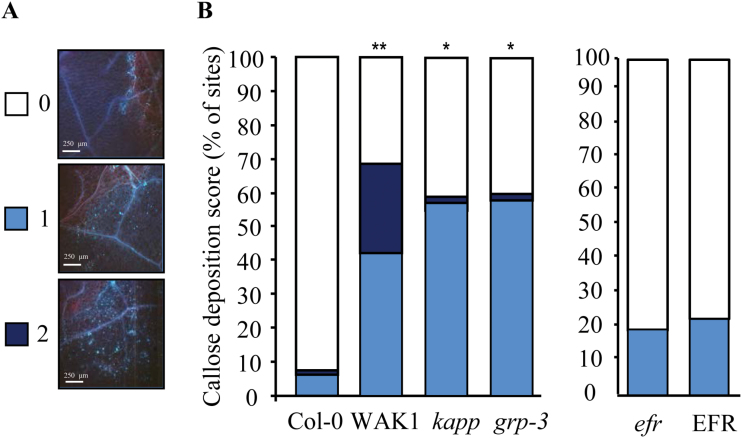
Transgenic plants overexpressing WAK1 and *grp-3* and *kapp* mutant plants show enhanced local response to wounding. Leaves were wounded by forceps and stained after 24h with aniline blue for callose visualization. Callose intensity in a region surrounding the wound site (the proximal region) was evaluated according to a score scale that varies between 0 (no deposition), 1 (a few deposits), and 2 (dense deposits). Representative callose deposition images for each score are shown in (A); all images are at the same scale. Bars, 250 μm. Histograms in (B) show the percentage of wound sites with a specific callose deposition score. Experiments were repeated five times (*n*=12) with similar results. Asterisks indicate statistically significant difference between control and transgenic plants, according to Fisher’s exact test (*, *P* <1×10^−3^; **, *P* <1×10^−4^).

OGs have been proposed to act as local signals in the response to wounding (Ryan and Jagendorf, 1995; Leon *et al.*, 2001; Savatin *et al.*, 2014*b*). Because WAK1-overexpressing plants and *grp-3* and *kapp* mutants all display an enhanced response to OGs, these plants are amenable to investigating the role of WAK1 and its interactors in the response to wounding. Callose deposition in response to mechanical damage inflicted by forceps was analysed in leaves in the proximity of the wounded tissue. Unlike wild-type plants characterized by callose deposition only at the very edge of the wound, transgenic WAK1 plants and *kapp* and *grp-3* mutants also showed callose deposition in a region surrounding the wound site up to a distance of 0.5–1mm from the edge of the wound (Fig. 4; see Supplementary Fig. S7A at *JXB* online). The response to wounding of transgenic EFR leaves, used as controls, was indistinguishable from that of wild-type leaves (Fig. 4).

**Fig. 5.**
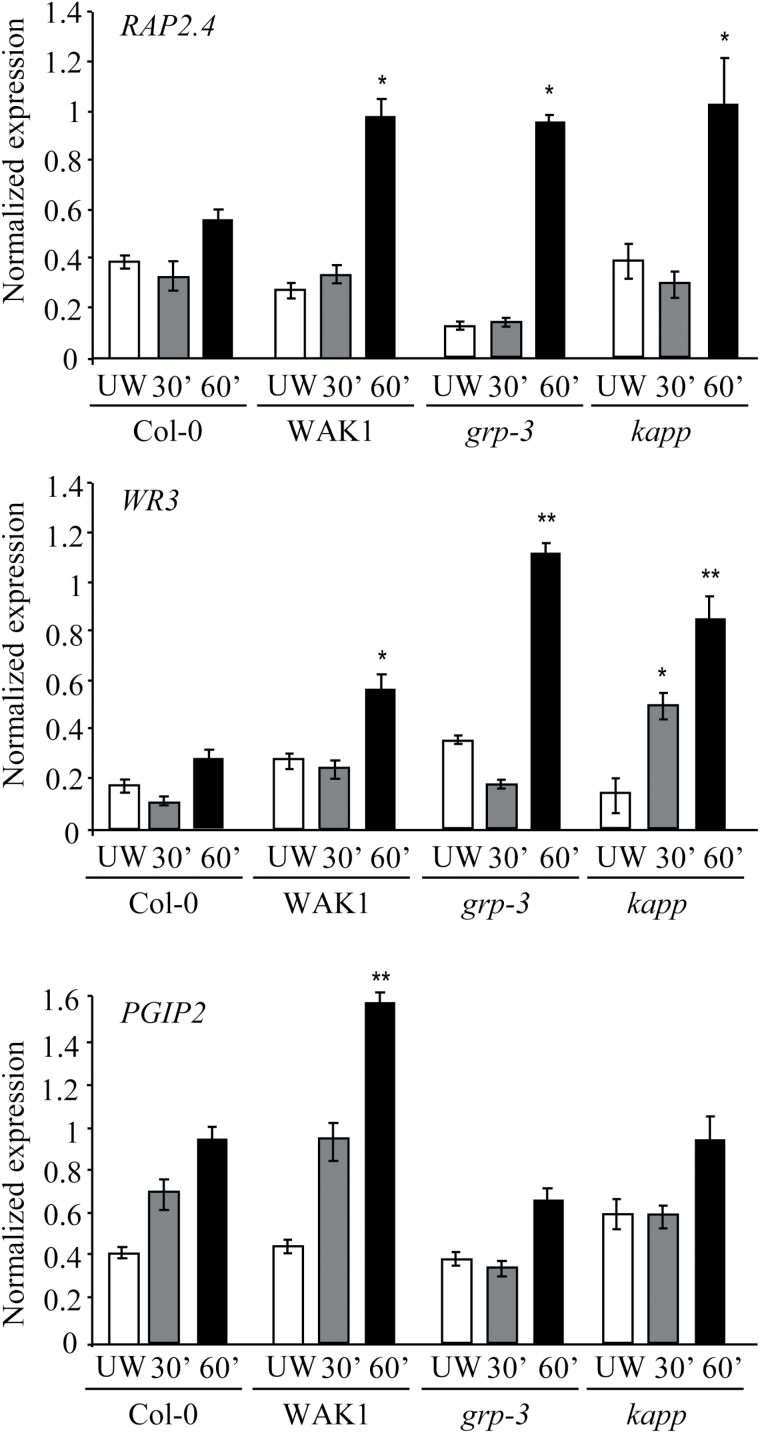
WAK1, *grp-3*, and *kapp* plants show an enhanced expression of wound-response genes in the area proximal to the wound site compared with the WT. Leaves of WAK1 (line #4), *grp-3*, and *kapp* plants were wounded by forceps and expression of *RAP2.4*, *WR3*, and *PGIP2* was analysed in unwounded leaves (UW) and in the area proximal to the wound site (Supplementary Fig. S7B) after 30 (grey bars) and 60min (black bars). Transcript levels were determined by Real-Time PCR, using *UBQ5* for normalization, and expressed as the gene/*UBQ5* ratio (normalized expression). Values are means ±SE of three independent experiments (*n*=6 in each experiment). Asterisks indicate statistically significant differences between corresponding treatments in Col-0 and each mutant/transgenic genotype, according to Student’s *t* test (*, *P* <0.05,**, *P* <0.01).

The expression of marker genes that are known to be expressed locally after wounding, namely *RAP2.4*, also known as *WIND1*, and encoding a member of the DREB subfamily A-6 of the ERF/AP2 transcription factor family (Delessert *et al.*, 2004), *WR3* encoding a high-affinity nitrate transporter (Titarenko *et al.*, 1997), and *PGIP2* (Ferrari *et al.*, 2003) also increased upon wounding in the proximal region (Supplementary Fig. S7B). In particular, expression of *RAP2.4* and *WR3* genes increased in WAK1, *kapp*, and *grp-3* plants as compared with Col-0 plants, whereas expression of *PGIP2* only increased in WAK1 leaves and did not increase in *kapp* and *grp-3* leaves (Fig. 5). Taken together, these data show that WAK1, the receptor of OGs, and its interactors GRP-3 and KAPP all play a role in the regulation of the local response to wounding.

### Loss of *GRP-3* and *KAPP* and overexpression of WAK1 lead to enhanced resistance to *Botrytis cinerea* but not to *Pectobacterium carotovorum*

**Fig. 6.**
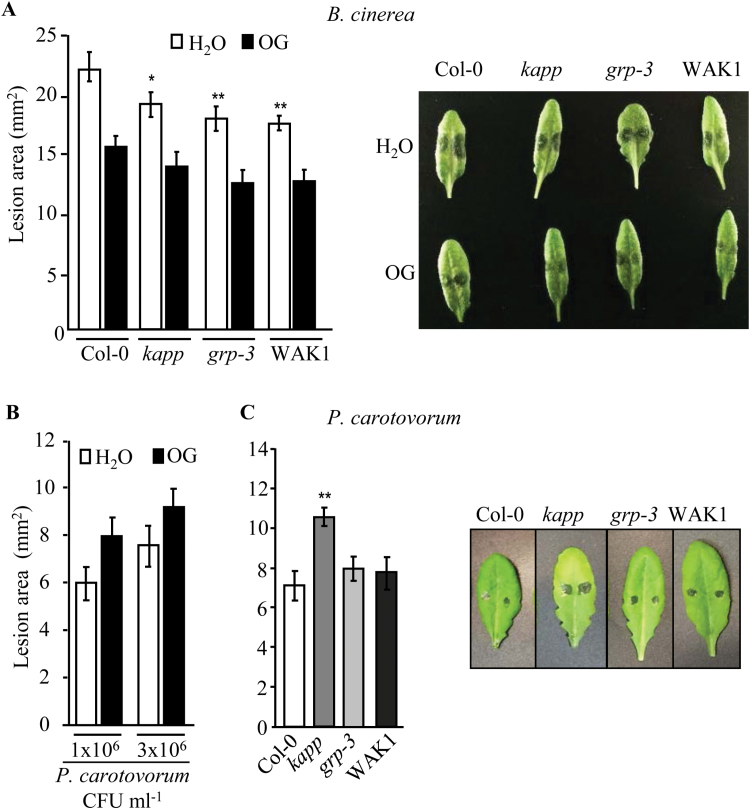
Loss of KAPP and GRP-3 and overexpression of WAK1 lead to enhanced basal resistance to *B. cinerea* but not to *P. carotovorum*. (A) Four-week-old wild-type, *grp-3, kapp*, and WAK1 plants were sprayed with water or 200µg ml^−1^ OGs and, after 24h, excised leaves were inoculated with *B. cinerea* (5×10^5^ conidiospores ml^−1^). Lesion size was measured at 48h post-infection. (B) Wild-type plants were sprayed with water or OGs as indicated in (A), and, after 24h, drop-inoculated with *P. carotovorum* cells at the indicated concentrations. (C) Wild-type, *grp-3, kapp*, and WAK1 plants were inoculated with *P. carotovorum* (3×10^6^ CFU ml^−1^). In both (B) and (C), lesion size was measured after 24h. In all infections, bars indicate average lesion area ±SE of at least two independent experiments (*n*=24, in each experiment). Asterisks indicate statistically significant differences against control (Col-0), according to Student’s *t* test (*, *P* <0.05; **, *P* <0.01). Representative images of infected leaves are shown.

The role of GRP-3 or KAPP in the response to pathogens has never been investigated, whereas *WAK1*, when overexpressed, has been reported to confer enhanced resistance to the necrotrophic fungus *B. cinerea* (Brutus *et al.*, 2010). We examined basal resistance to *B. cinerea* as well as OG-induced protection in *kapp* and *grp-3* plants and, in parallel, in WAK1 plants, by spraying plants with either water or OGs and then by inoculating excised leaves with *B. cinerea* spores after 24h. Development of lesions at 48h in water-treated leaves was lower than in the wild type, by about 15% in the *kapp* mutant and about 20% in both the *grp-3* mutant and the WAK1 plants. OG pretreatment increased resistance to the fungus in all plants in a similar manner (Fig. 6A).

Response to the necrotrophic bacterium *P. carotovorum* was also analysed (Davidsson *et al.*, 2013). Because it is not known whether pretreatment with OGs confers protection against this pathogen, we first examined the OG-induced protection response in wild-type plants. No protection against *P. carotovorum* was observed (Fig. 6B). Next, adult WAK1 and mutant plants were drop-inoculated with *P. carotovorum* and the lesion area was measured after 24h. Disease symptoms were similar to the wild type in WAK1 and *grp-3* plants, whereas they were higher in *kapp* plants (Fig. 6C).

### Localization of KAPP and GRP-3 is consistent with a functional interaction with WAK1

A requisite for the physical and functional interaction between WAK1, GRP-3, and KAPP is their co-localization in the cell. Localization of KAPP and WAK1 on the plasma membrane has previously been reported (Shah *et al.*, 2002; Brutus *et al.*, 2010), whereas localization of GRP-3 is unknown. The *GRP-3*-encoded product exhibits a putative N-signal peptide (von Heijne, 1988) and no other membrane-spanning domain or canonical organelle retention signal, suggesting a cell wall localization. The localization of GRP-3 and, in parallel, of KAPP was investigated by the stable expression of fluorescent forms of the proteins in Arabidopsis transgenic plants.

**Fig. 7.**
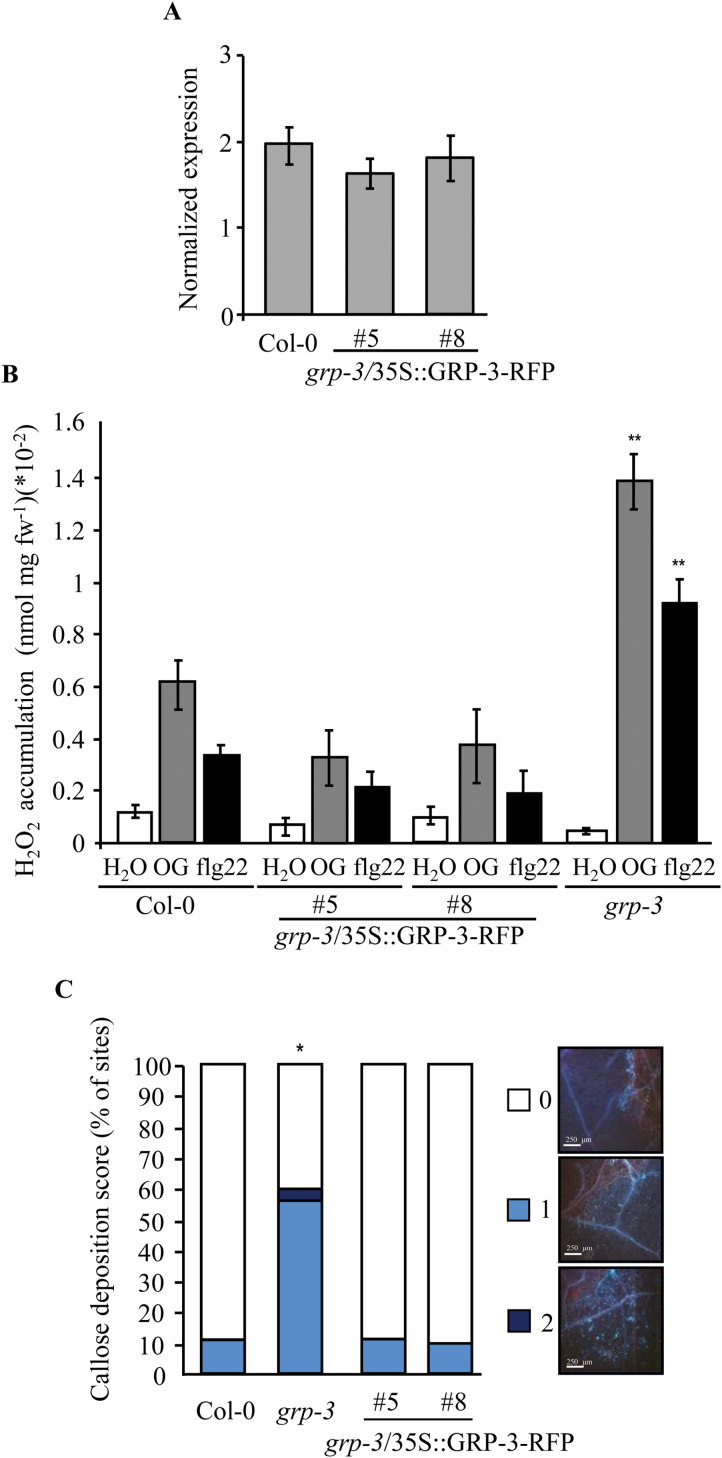
The GRP-3-RFP fusion complements the altered phenotype of the *grp-3* mutant. (A) Expression of *GRP-3* in 10-d-old seedlings of the wild type (Col-0) and two independent transgenic lines stably transformed with a construct for the expression of a GRP-3-RFP fusion under the control of the 35S promoter (*grp-3/*35S::GRP-3-RFP plants, lines #5 and #8). Expression was determined by qRT-PCR using *UBQ5* for normalization. Results are expressed as normalized expression (gene/*UBQ5*). Values are the mean (±SE) of three independent experiments (*n*=20). (B) Fourteen-day-old untransformed *grp-3* and transgenic *grp-3*/35S::GRP-3-RFP lines were treated with water (white bars), OGs (50 μg ml^−1^, grey bars), and flg22 (100nM, black bar,) and the accumulation of H_2_O_2_ was measured by xylenol orange assay. Results are means of three independent experiments (±SE; *n*=40 in each experiment). Asterisks indicate statistically significant difference between control and transgenic plants, according to Student’s *t* test (*, *P* <5×10^−4^; **, *P* <5×10^−6^). fw, fresh weight. (C) Leaves of untransformed *grp-3* and transgenic *grp-3*/35S::GRP-3-RFP lines were wounded by forceps and stained after 24h with aniline blue for callose visualization. Callose intensity in the wound proximal region was expressed by different score as indicated. Representative callose deposition for each score is shown on the right; all images are at the same scale, bars 250 μm. The histograms on the left show the percentage of wound sites with a specific callose deposition score. Experiments were repeated three times (*n*=12) with similar results. Asterisks indicate statistically significant difference between control and transgenic plants, according to Fisher’s exact test (*, *P* <1×10^−4^).

A fusion of GRP-3 with the Red Fluorescent Protein (RFP) driven by the CaMV 35S promoter (35S::GRP-3-RFP) was expressed in the *grp-3* mutant as well as in the wild-type background in order to assess whether the fusion is functional and, at the same time, prove that the defective phenotype of the *grp-3* mutant is indeed due to the lack of *GRP-3.* Among six independent *grp-3* transformed lines, two (*grp-3*/35S::GRP-3-RFP, #5 and #8) were selected that carried a single insertion of the transgene and showed levels of *GRP-3* transcripts comparable with those of the wild type, as determined by qRT-PCR (Fig. 7A), and brought to homozygosis. Similarly, among seven independent transformed lines expressing GRP-3-RFP in the wild-type background, three homozygous transgenic wild-type lines carrying a single insertion of the transgene were obtained. Compared with those of the wild type, levels of *GRP-3* (endogenous+transgene) transcripts were higher in lines 35S::GRP-3-RFP #16 and #17, hereon indicated as GRP-3-OE plants, and similar in line #4, hereon indicated as GRP-3 #4 plants (Supplementary Fig. S2D). The fluorescent protein was clearly visible in all of the selected transgenic plants as determined by confocal microscopy (see below), except for the GRP-3 #4 plants in which fluorescence was barely detectable.

The functionality of the fusion and its capability of complementing the mutant phenotype was analysed first by testing *grp-3/*35S::GRP-3-RFP seedlings for H_2_O_2_ production after treatment with OGs, flg22, and water. Treatment with both elicitors induced levels of H_2_O_2_ similar to those of the wild type and significantly lower than those observed in the *grp-3* mutant (Fig. 7B). Next, callose deposition in response to wounding was examined. Callose deposition in the complemented transgenic lines 5 and 8 was similar to that of wild type, i.e. it was limited to the very edge of the wound and not to the surrounding cells (Fig. 7C). These data indicate that the GRP-3-RFP fusion protein is functional and complements the *grp-3* mutant to normal H_2_O_2_ accumulation after elicitor treatment and to normal callose deposition upon wounding, supporting our conclusion that the presence of GRP-3 negatively affects immunity.

**Fig. 8.**
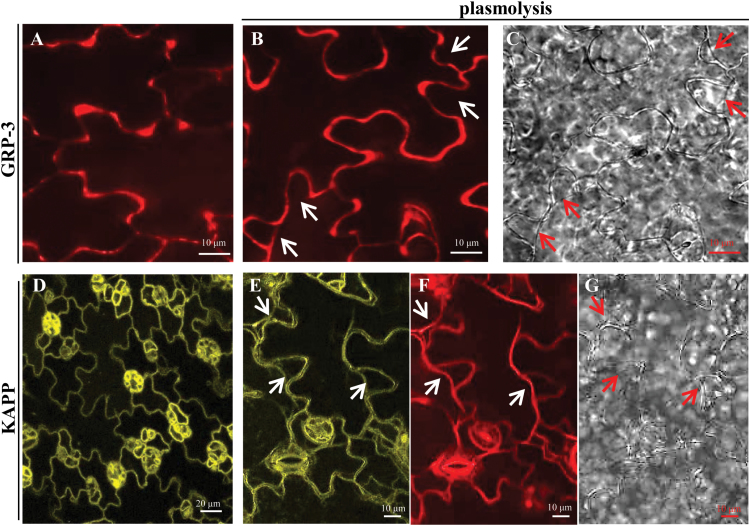
Localization of GRP-3 and KAPP in epidermal cells of Arabidopsis seedlings. Ten-day-old wild-type transgenic seedlings stably expressing GRP-3-RFP (A) and KAPP–YFP (D). (B, C, E–G) Localization upon plasmolysis induced by 800mM mannitol for 20min. (B) GRP-3-RFP; fluorescence associated with the retracted plasma membrane is shown by the arrow at the bottom. (C) Bright field. (E) KAPP–YFP. (F) staining with the plasma membrane-specific dye FM4-64. (G) Bright field. Analyses were performed by spinning disc confocal microscopy (B, C) and laser scanning confocal microscopy (E–G). In (B) and (C), and (D–G), arrows indicate sites where the plasma membrane is detached from the cell wall. Images are representative of two independent transgenic lines for each construct. Transgenic *grp-3*/35S::GRP-3-RFP showed a fluorescence pattern similar to that shown in (A).

Confocal microscopy analysis of epidermal cells of Arabidopsis *grp-3* and wild-type plants expressing GRP-3-RFP showed a pattern of fluorescence that was similar and indicative of localization at the cell periphery, probably in the apoplast (Fig. 8A). Upon plasmolysis induced by 800mM mannitol, a strong fluorescence signal not associated with the retracted membranes was observed, confirming a cell wall localization; a weak fluorescence, however, was associated with some retracted area of the plasma membrane (Fig. 8B, C), suggesting that the protein also interacts with the plasma membrane, possibly through interaction with receptors.

A Yellow Fluorescent Protein (YFP)–KAPP fusion driven by the CaMV 35S promoter was expressed in wild-type plants. Among nine independent transformed lines, two homozygous single-insertion (#7 and #10; hereon indicated as KAPP-OE plants) were obtained, showing higher *KAPP* transcript levels compared with the wild type (Supplementary Fig. S2D). Fluorescent KAPP was mainly localized on the plasma membrane (Fig. 8D). Plasmolysis and co-localization with the plasma membrane specific dye FM4-64 (Brandizzi *et al.*, 2004) showed an association of KAPP fluorescence to the retracted membranes (Fig. 8E, F, G), possibly through the interaction with plasma membrane receptors such as WAK1 or FLS2. This result confirms the plasma membrane localization previously reported by Shah *et al.* (2002).

### Elicitor-induced gene expression is negatively affected by KAPP, but differentially affected by GRP-3

**Fig. 9.**
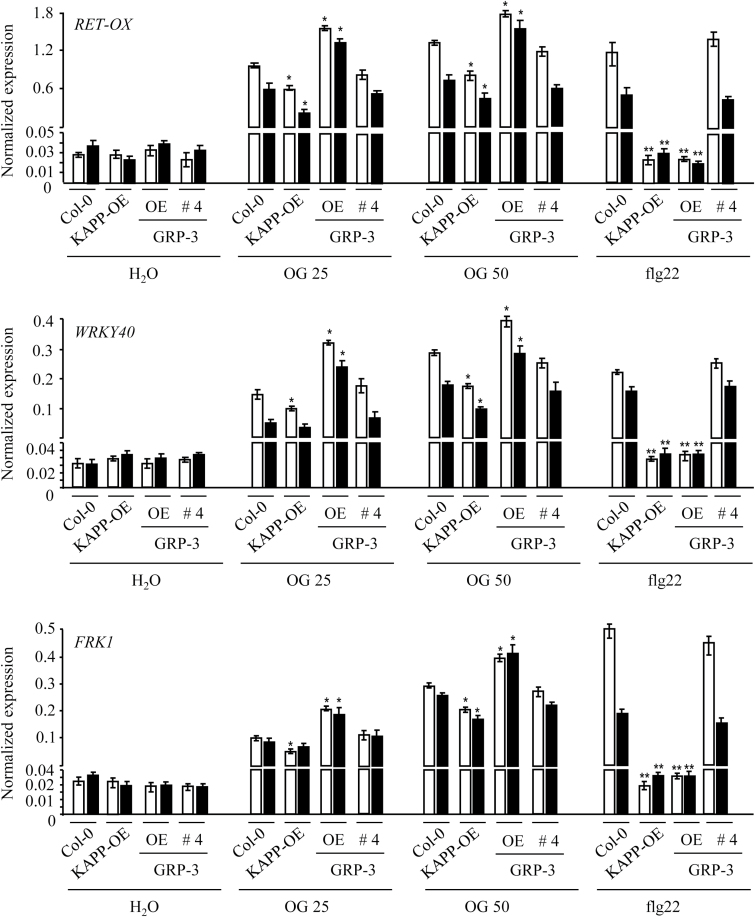
Expression of defense response genes induced by elicitors in seedlings overexpressing *KAPP* and *GRP-3*. Seedlings of the lines overexpressing KAPP and GRP-3 [KAPP-OE (line #7) and GRP-3-OE (line #17)] and of the Col-0/35S::GRP-3-RFP line #4, which exhibits *GRP-3* transcript levels similar to those of the wild type (Supplementary Fig. S2D) and, therefore, represents a negative control, were treated with water, OGs (25 and 50 μg ml^−1^), and flg22 (10nM) and accumulation of *RET-OX*, *WRKY40*, and *FRK1* transcripts was analysed after 1h (white bars) and 3h (black bars) by qRT-PCR, using *UBQ5* for normalization. Transcript levels are expressed as the gene/*UBQ5* ratio (normalized expression). Values are mean (±SE) of two independent experiments (*n*=20, in each experiment). Asterisks indicate statistically significant differences between elicitor treatment of overexpressing seedlings and Col-0, according to Student’s *t* test (*, *P* <0.05; **, *P* <1×10^−3^).

The response to OGs and flg22 was analysed in the KAPP-OE, GRP-3-OE, and GRP-3 #4 plants. The expression of *Ret-Ox*, *WRKY40*, and *FRK1* in seedlings was examined upon treatment with the elicitors for 1h and 3h. In the KAPP-OE plants, the expression of the three genes was significantly lower than in the wild type at both time points and at the two concentrations of OGs (25 and 50 μg ml^−1^), and was not induced by flg22, confirming a negative role of *KAPP* in elicitor-induced immunity (Fig. 9; see Supplementary Fig. S8 at *JXB* online). Notably, in GRP-3-OE seedlings, the expression of the three genes did not increase in response to flg22 and was induced to a higher extent compared with the wild type at both OG concentrations and at both time points (Fig. 9; Supplementary Fig. S8). GRP-3 #4 seedlings behaved like the wild type (Fig. 9). As in Col-0 seedlings, no induction of gene expression of the three genes was observed after treatment with OG3 in KAPP-OE, GRP-3-OE, and GRP-3 #4 lines (Supplementary Fig. S8; see Supplementary Fig. S9 at *JXB* online). As in the case of the loss-of-function mutants (Fig. 1), basal expression of the genes was comparable with that in the wild type.

Production of extracellular hydrogen peroxide was also analysed after treatment with both OGs and flg22 in KAPP-OE and GRP-3-OE seedlings. Both elicitors induced levels of H_2_O_2_ that were similar to those of the wild type in all the lines examined (see Supplementary Fig. S10 at *JXB* online).

## Discussion

Pectin is one of the first cell wall structures to be attacked during pathogen invasion (De Lorenzo *et al.*, 2011; Lionetti *et al.*, 2012; Bellincampi *et al.*, 2014). Monitoring the pectin status is probablyy critical in the control of cell wall integrity. OGs are possible indicators of an impairment of the cell wall integrity both in physiological and pathological conditions (De Lorenzo *et al.*, 2011; Hamann, 2012; Malinovsky *et al.*, 2014). OGs are well known DAMPs, released upon partial degradation of homogalacturonan by microbial pectic enzymes in pathological conditions (Ferrari *et al.*, 2013; Benedetti *et al.*, 2015) and by plant-derived enzymes in physiological conditions (Patterson, 2001; Roberts *et al.*, 2002; Gonzalez-Carranza *et al.*, 2007; Ogawa *et al.*, 2009; Xiao *et al.*, 2014; Pontiggia *et al.*, 2015). It has been demonstrated that WAK1 is a receptor of OGs (Brutus *et al.*, 2010). With its ability to sense OGs, WAK1 appears to be a key element in the communication to the cell of an altered state of the wall. WAK1 forms a complex with two proteins, a glycine rich protein (GRP-3) that is demonstrated in this paper to be localized in the apoplast and, in small amounts, on the plasma membrane probably on the apoplastic side, and a membrane-associated cytosolic phosphatase (KAPP) (Park *et al.*, 2001). GRP-3 is a 122-amino acid protein, in its mature form, and its Cys-rich C-terminal portion (34 amino acids) has been proposed to be essential for the interaction with WAK1 (Park *et al.*, 2001). KAPP is a larger protein composed of three domains: an amino-terminal signal anchor, a kinase interaction (KI) domain, and a type 2C protein phosphatase catalytic region. It has been previously described as a negative regulator of response to MAMP (Gomez-Gomez *et al.*, 2001). Consistently, plants overexpressing KAPP do not respond to flg22 like the *fls2* mutant (Gomez-Gomez *et al.*, 2001).

In this study, using loss-of-function mutants, we show that not only *KAPP* but also *GRP-3* plays a negative role in the response to OGs and flg22. Moreover, we show that *grp-3* and *kapp* plants, as well as WAK1 plants, are more resistant to *B. cinerea* (Brutus *et al.*, 2010), supporting the vision that OG signalling plays an important role in this pathosystem (Mengiste, 2012). By contrast, the three plants are not more resistant to *P. carotovorum*, in agreement with the observation that pretreatment with OGs does not confer any protection against this bacterium. This observation suggests that the enhanced levels of OGs present in the OGM plants are unlikely to explain the increased resistance against *P. carotovorum* (Benedetti *et al.*, 2015). Short OGs, which are not sensed through the WAK1/GRP-3/KAPP perception system (this work), confer protection against this bacterium and have been proposed to play a more important role in resistance against bacterial necrotrophs and herbivores compared with the longer OGs (Davidsson *et al.*, 2013, and references therein). Short OGs are present at increased levels in the OGM plants compared with wild-type plants and may be responsible for the enhanced resistance of these plants to *P. carotovorum*. Longer OGs may instead play a defensive role mainly against necrotrophic fungi.

On the other hand, whereas the analyses performed with the overexpressing plants confirm the negative role of KAPP in both flg22 and OG signalling, they point to a different and more complex role of GRP-3 in the response to OGs. While the loss of *GRP-3* appear to prolong the duration of the expression of defence response genes, the overexpression of this protein leads to an enhanced gene expression. GRP-3 has been reported to be necessary for the binding of KAPP to WAK1, suggesting that GRP-3 induces on WAK1 a conformational change required for the formation of the KAPP/WAK1 complex. If the binding of GRP-3 to WAK1 increases the affinity of the receptor to the OGs, GRP-3 may act as an allosteric modulator through a mechanism that has been recognized to occur generally for the control of receptor function (Tsai and Nussinov, 2014). Modulators bind to regulatory sites distinct from the active site on the receptor, resulting in conformational changes that may profoundly influence protein function. The strong negative effect of *GRP-3* overexpression on flg22 signalling is unexpected and its mechanistic bases are not obvious. Publicly available transcriptome data indicate that *GRP-3* is hardly induced during the immune response or by hormones, suggesting that its transcript levels are maintained quite constant. Moreover, a jasmonate-dependent >2-fold decrease of GRP-3 in the proximal zone of the 2.5-mm wound at 6h after injury was shown in a proteomic study (Gfeller *et al.*, 2011), indicating that, at the protein level, GRP-3 is down-regulated rather than up-regulated in the wound response, probably to ensure an appropriate duration of the response to many different danger signals. Thus, whether this action of diverting the immune response towards OG signalling at the expenses of MAMP signalling has a biological significance remains to be elucidated.

The absence of *KAPP* and *GRP-3* does not appear to affect the initial sensing event, whereas it influences the secondary phase of the response to elicitors. In agreement with a role in a secondary phase of the response to elicitors, a late response such as callose deposition is affected by the loss of *KAPP* and *GRP-3*. An enhanced callose deposition is observed in the *grp-3* and *kapp* mutant leaves in response to sprayed flg22 and elicitor-active OGs, but not to a short OG. Notably, an enhanced response to elicitor-active OGs, but not to flg22 or the elicitor-inactive OGs, is exhibited by transgenic plants overexpressing WAK1. Adult EFR transgenic plants, used as controls, showed an enhanced response only to elf18 and not to OGs. WAK1-overexpressing plants also showed enhanced ROS production in response to OGs, but not to flg22. Thus, in both types of transgenic plants overexpressing a PRR, the response was ligand-specific, probably as a direct consequence of the receptor overexpression, and not of secondary or compensatory effects such as perturbation of the plasma membrane sensing capability of the transgenic plants. In this regard, it is worth noting that plants overexpressing WAK1 and EFR and *grp-3* mutant plants show a slight increase of callose deposition in leaves sprayed with water alone, although the ligand-specific response to elicitors far exceeds the response to mock treatment, suggesting that these plants may have a generally higher sensitivity to spraying or stress stimuli.

Unexpectedly, the response of WAK1 seedlings to OGs was not significantly different from that of wild-type seedlings. This may be due to the lower expression of the transgene in the seedlings compared with leaves; alternatively, the perception/transduction system for OGs is already at saturation in the young and developing tissues of the seedlings and is not amenable to further enhancement.

We found that *WAK1*, *GRP-3*, and *KAPP*, which all play a role in the response to OGs, also play a role in the local response to wounding. Wounding of plant tissues offers an ideal entry point for many pathogenic microbes. Plants have evolved mechanisms to sense and respond to wounding by activating the proper defences against invading micro-organisms as well as to insects (Bradley *et al.*, 1992; Chang *et al.*, 1995; Reymond *et al.*, 2000; Heil and Land, 2014; Savatin *et al.*, 2014*b*). Since WAK1 is an OG receptor, its involvement in the response to wounding supports the hypothesis that OGs act as local signal molecules. On the other hand, GRP-3 and KAPP proteins, that are localized to the apoplastic and the cytosolic side of the plasma membrane, respectively, act as negative regulators of both OG-activated signalling cascade and wound response. The role of *KAPP* and *GRP-3* as negative regulators of the immune response is likely to be important in plant growth. After being triggered, the immune system needs to return to the baseline at the appropriate time so as not to become as deleterious as the inciting stress stimulus. The mechanisms underlying the phasing out of the plant immune response have hardly been explored, and here we show that GRP-3 and KAPP may function in restoring the pre-damaged state.

## Supplementary data

Supplementary data can be found at *JXB* online.

Table S1. Primer sequences used in the characterization of mutant lines and to generate the constructs.

Table S2. Primer sequences used in gene expression analysis.

Figure S1. *grp-3* and *kapp* mutant lines are null mutants.

Figure S2. Analyses of transcript levels in transgenic plants.

Figure S3. A second independent insertion mutant for KAPP (*kapp-2*) shows behaviour similar to that of the *kapp* mutant.

Figure S4. Arabidopsis seedlings overexpressing WAK1 do not show alteration in the OG-induced expression of defence response genes.

Figure S5. A second independent line of Arabidopsis overexpressing WAK1 shows behaviour similar to that of line WAK1 #4.

Figure S6. A transgenic line transformed with the empty vector does not show enhanced OG-induced callose deposition and enhanced local response to wounding.

Figure S7. WAK1 plants show enhanced local response to wounding.

Figure S8. Marker gene expression analysis in response to elicitors in seedlings of second independent lines overexpressing *KAPP* and *GRP-3*.

Figure S9. Treatment with short OGs do not induce expression of defence response genes in Arabidopsis seedlings overexpressing *KAPP* and *GRP-3*.

Figure S10. Elicitor-induced production of extracellular hydrogen peroxide in KAPP and GRP-3 overexpressing seedlings.

Supplementary Data
