# Supplementary material for: GRP-3 and KAPP, encoding interactors of WAK1, negatively affect defense responses induced by oligogalacturonides and local response to wounding
Source: J Exp Bot. 2016 Jan 8;67(6):1715–29. doi: 10.1093/jxb/erv563 (PMC4783359; doi:10.1093/jxb/erv563)
Supplement: Supplementary Data [file supp_67_6_1715__index.html]

 GRP-3 and KAPP, encoding interactors of WAK1, negatively affect defense responses induced by oligogalacturonides and local response to wounding — GRP-3 and KAPP, encoding interactors of WAK1, negatively affect defense responses induced by oligogalacturonides and local response to wounding — Supplementary Data 

# *GRP-3* and *KAPP,* encoding interactors of WAK1, negatively affect defense responses induced by oligogalacturonides and local response to wounding

## Supplementary Data

Data files

- supplementary\_figures\_S1\_S10\_tables\_S1\_S2.pdf - Supplementary Data
