# Supplementary material for: GRP-3 and KAPP, encoding interactors of WAK1, negatively affect defense responses induced by oligogalacturonides and local response to wounding
Source: J Exp Bot. 2016 Jan 8;67(6):1715–29. doi: 10.1093/jxb/erv563 (PMC4783359; doi:10.1093/jxb/erv563)
Supplement: Supplementary Data [file supp_erv563_supplementary_figures_S1_S10_tables_S1_S2.pdf]

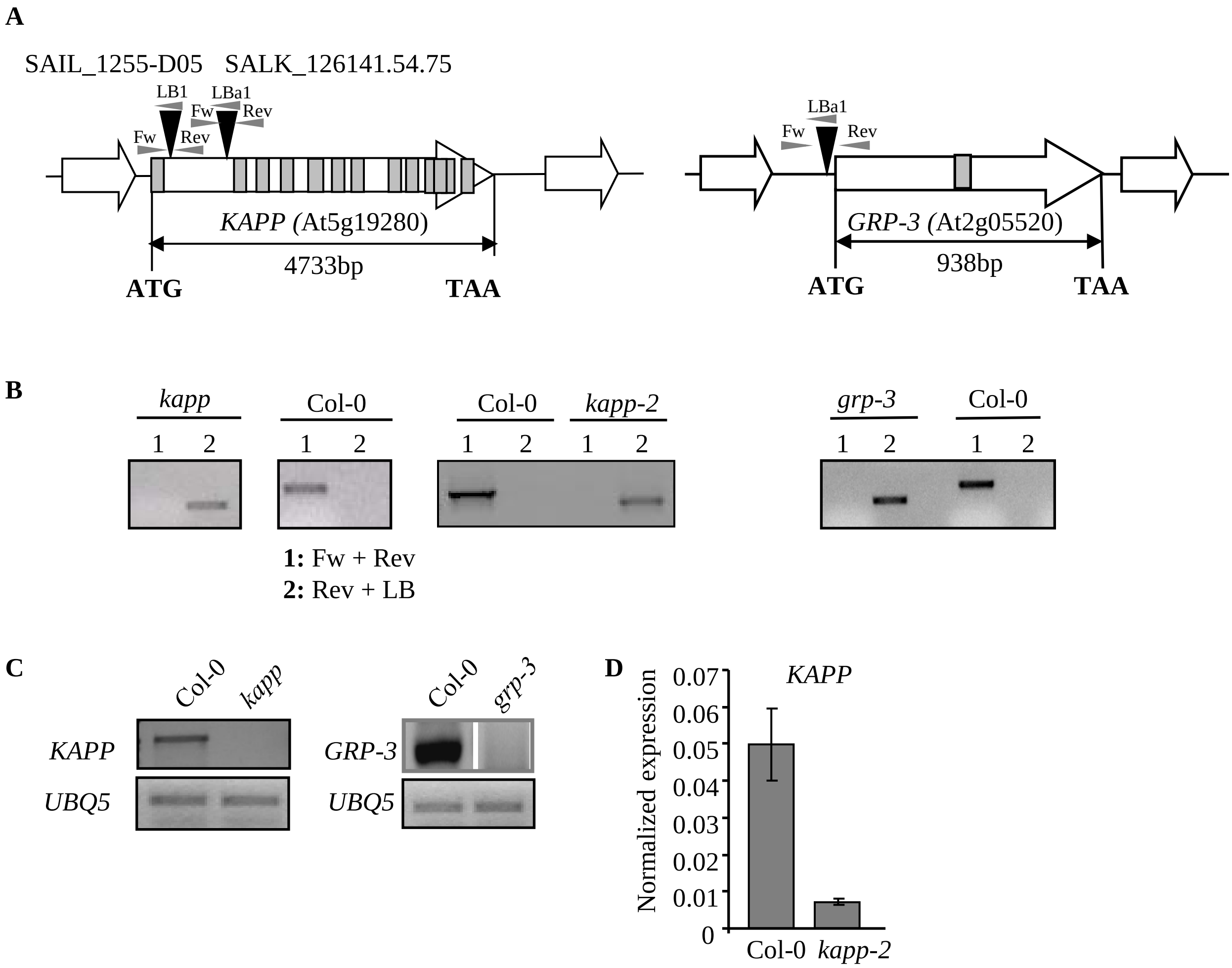

Supplementary Figure S1. *grp-3* and *kapp* mutant lines are null mutants. (A) In both the *kapp* and *kapp-2* allelic mutants (SAIL_1255-D05 and SALK_126141.54.75, respectively, both in Col-0 background) the T-DNA insertion site is in the first intron of the gene. In the *grp-3* mutant (SALK_084685.46.60, Col-0 background), the T-DNA insertion site is within the 5'UTR (black arrowhead). Genes, including the neighbor ones, are shown as block arrows with arrowheads indicating the 3' terminus. Exons are indicated as grey boxes. The gray arrowheads indicate the forward (Fw), the reverse (Rev) and the left-border (LBa1 for the SALK lines and LB1 for the SAIL line) primers used for diagnostic PCR (shown in B). (B) PCR-based genotyping using gene specific PCR primers (listed in Supplementary Table 1), performed to select homozygous mutants. (C) Analysis of *GRP-3* and *KAPP* transcripts was performed by RT-PCR in wild-type (Col-0), *grp-3* and *kapp* seedlings. *UBQ5* was analysed as a control to show that equal amounts of cDNA were used. Mutants are homozygous for the insertions, which functionally disrupt the expression of the *GRP-3* and *KAPP* gene. (D) Analysis of *KAPP* transcript was performed in *kapp-2* seedlings by qRT-PCR using *UBQ5* for normalization; results are expressed as Normalized expression (gene/*UBQ5*). Values are the mean (± SE) of three independent experiments (n = 20 in each experiment).

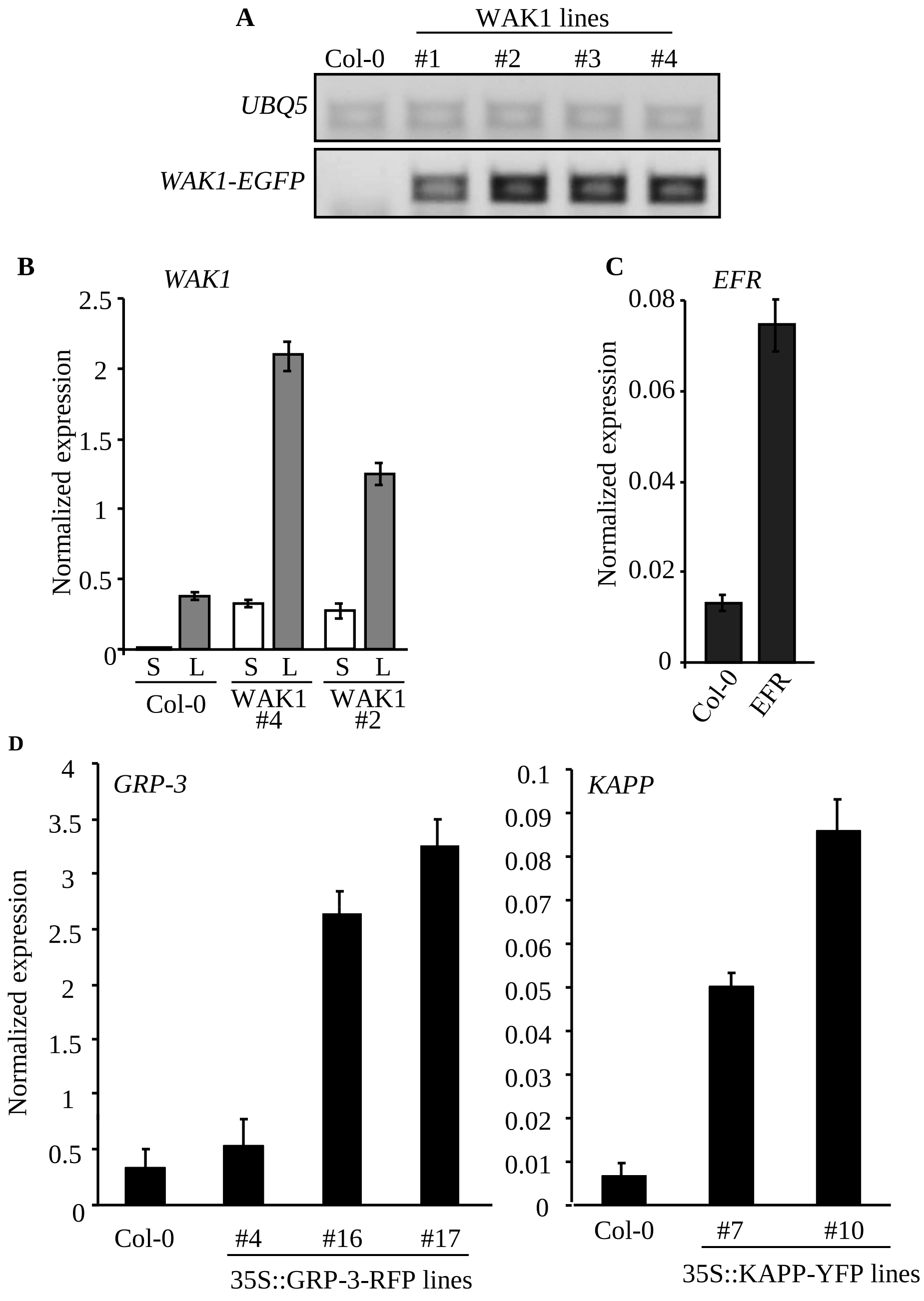

Supplementary Figure S2. Analyses of transcript levels in transgenic plants. (A) Transcript levels of the *WAK1-EGFP* transgene were analyzed by RT-PCR, using specific primers that amplify a fragment encompassing the junction between *WAK1* and *EGFP*, in T2 seedlings of independent WAK1 lines. *UBQ5* was analyzed to show that equal amounts of cDNA were used**.** (B) The expression of the *WAK1* gene (endogenous + transgene) was analyzed in wild-type and WAK1 overexpressor (lines #2 and #4) seedlings (S) and leaves (L) using primers specific for the region encoding the *WAK1* ectodomain. WAK1 transcript level in wild-type seedlings was 0.007 ± 0.0015. (C) Levels of *EFR* transcripts (endogenous + transgene) was examined in adults leaves of Col-0 and EFR plants using primers specific for the region encoding the EFR ectodomain. (D) The expression of the indicated genes was analyzed in seedlings of the independent transgenic lines 35S::GRP-3-RFP and 35S::KAPP-YFP examined in this work. In B, C and D analyses were performed by qRT-PCR using *UBQ5* for normalization and results are expressed as normalized expression (gene/*UBQ5*). Values are the mean (± SE) of three independent experiments (for seedlings and leaves n = 20 and n = 5, respectively, in each experiment).

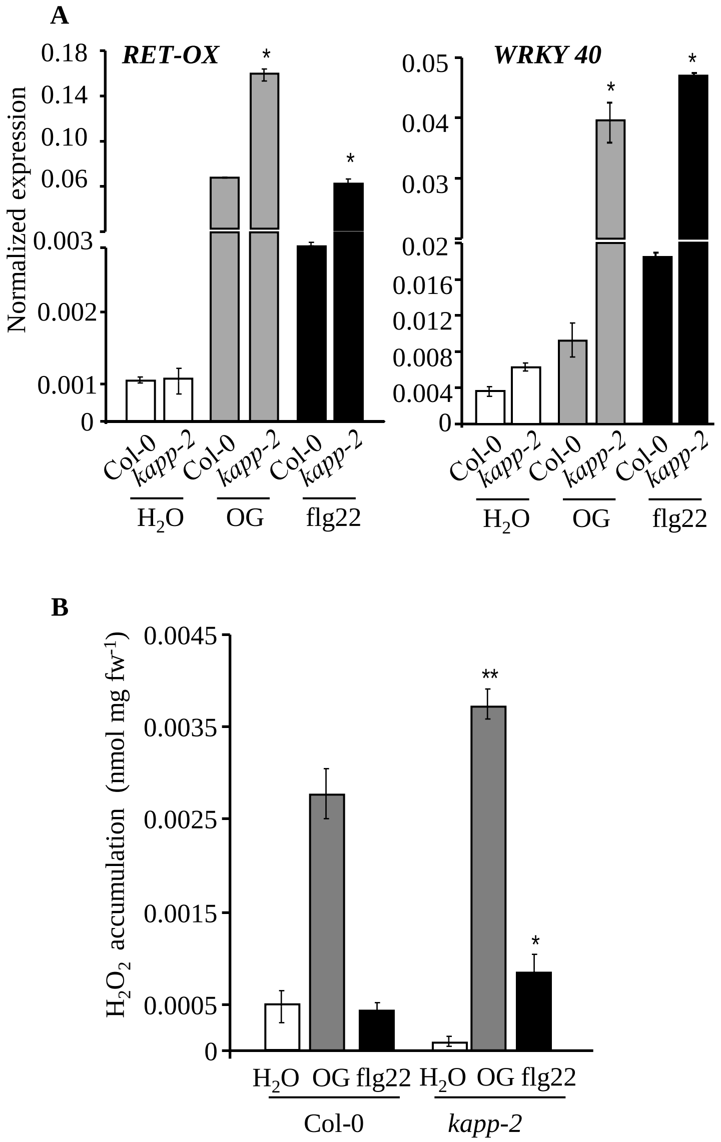

Supplementary Figure S3. A second independent insertion mutant for KAPP (*kapp-2*) shows a behavior similar to that of *kapp* mutant. (A) Seedlings were treated with OGs (50 µg ml^{-1}, gray bars) or flg22 (10 nM, black bars) or water (white bars), as a control, and accumulation of *RET-OX* and *WRKY40* transcripts was analyzed after 3 h by qRT-PCR, using UBQ5 for normalization. Transcript levels are expressed as the gene/UBQ5 ratio (normalized expression). Values are means (± SE) of three independent experiments (n = 20, in each experiment). Asterisks indicate statistically significant differences between elicitor treatment of mutant seedlings and Col-0, according to Student's t test (*, $p < 0.001$). (B) *kapp-2* seedlings were treated with water (white bar), OGs (50 µg ml^{-1}, gray bar) and flg22 (100 nM, black bar) and accumulation of H_2O_2 was measured by xylenol orange assay. Results are means of three independent experiments (± SE; n = 40 in each experiment). Asterisks indicate statistically significant difference between control and transgenic plants, according to student T-test (* $p < 0.05$; ** $p < 0.01$). fw, fresh weight.

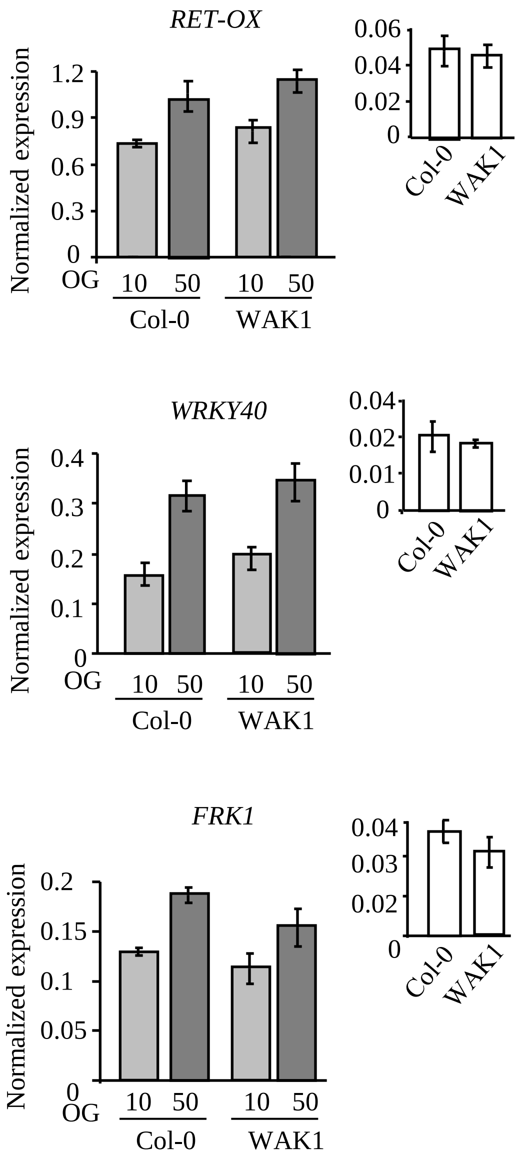

Supplementary Figure S4. Arabidopsis seedlings overexpressing WAK1 do not show alteration in the OG-induced expression of defense response genes. Col-0 and WAK1 seedlings (line #4) were treated with water (white bars in inset) or OGs (10 and 50 µg ml^{-1}, light gray bar and dark gray bar, respectively) for 1 h, and accumulation of *RET-OX*, *WRKY40* and *FRK1* transcripts was analyzed by qRT-PCR, using *UBQ5* for normalization. Results are expressed as gene/*UBQ5* (normalized expression) and are means (± SE) of three independent experiments (n = 20 in each experiment). No statistically significant differences between OG treatment of Col-0 and WAK1 seedlings were observed, according to Student's t-test. Similar results were obtained using seedlings of the WAK1 transgenic line #2.

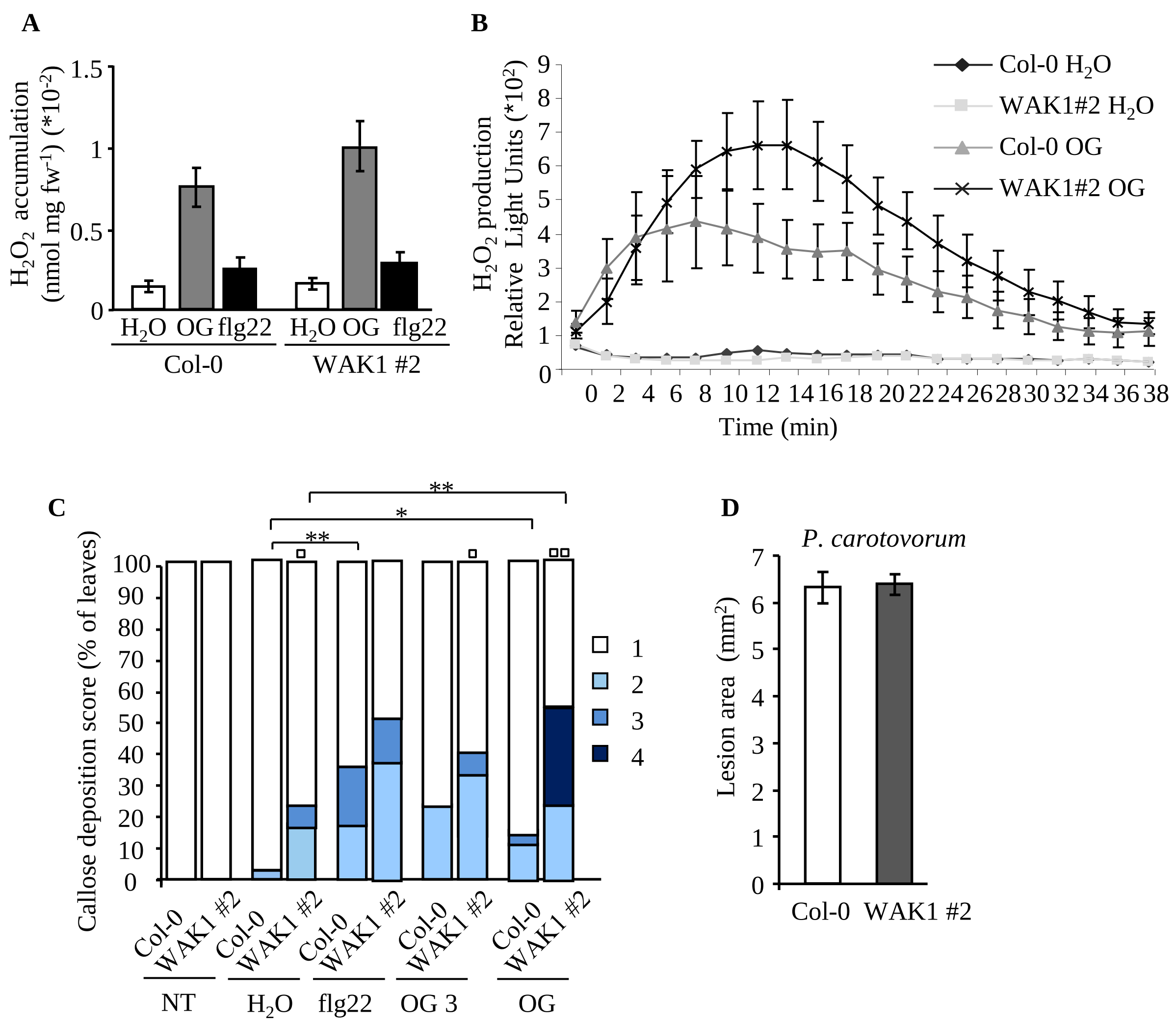

Supplementary Figure S5. A second independent line of Arabidopsis overexpressing WAK1 shows a behavior similar to that of line WAK1 #4. (A) WAK1 (line #2) seedlings were treated with water (white bar), OGs (50 μg ml^{-1}, gray bar) and flg22 (100 nM, black bar) and accumulation of H_2O_2 was measured by xylenol orange assay. Results are means of four independent experiments (± SE; n = 40 in each experiment). No statistically significant differences between elicitor treatment of Col-0 and WAK1 #2 seedlings were observed, according to Student's t-test. (B) Hydrogen peroxide production was measured using a luminol-based assay in leaf discs from WAK1 line #2 and Col-0 plants after elicitation with water and OGs (150 μg ml^{-1}) or flg22 (10 μM). Results are mean ± SE of three independent experiments (n = 12). (C) Callose deposition in WAK1 #2 leaves sprayed with H_2O or elicitors (flg22, 100 nM; OG 3, 50 μg ml^{-1}; OG, 50 μg ml^{-1}) and stained after 24 h with aniline blue. Callose deposition is expressed as a score as indicated in panel A of Fig. 3. The histograms show the percentage of leaves with a specific callose deposition score. White squares directly above bars indicate statistically significant difference between Col-0 plants and transgenic plants. Asterisks above connection lines indicate statistically significant difference between water and elicitors treatment in each background plants, according to Fisher's exact test (* or white square $p < 0.05$; ** $p < 0.005$; *** $p < 1 \times 10^{-4}$). Five independent experiments were performed (n = 12 in each experiments). (D) WAK1 (line #2) leaves were inoculated with *P. carotovorum* (3 x 10^6 CFU/ml) and lesion area was analyzed after 16 h. Values are means ± SE of at least 16 lesions. Asterisks indicate statistically significant differences against control (Col-0), according to Student's t-test (*$p < 0.05$).

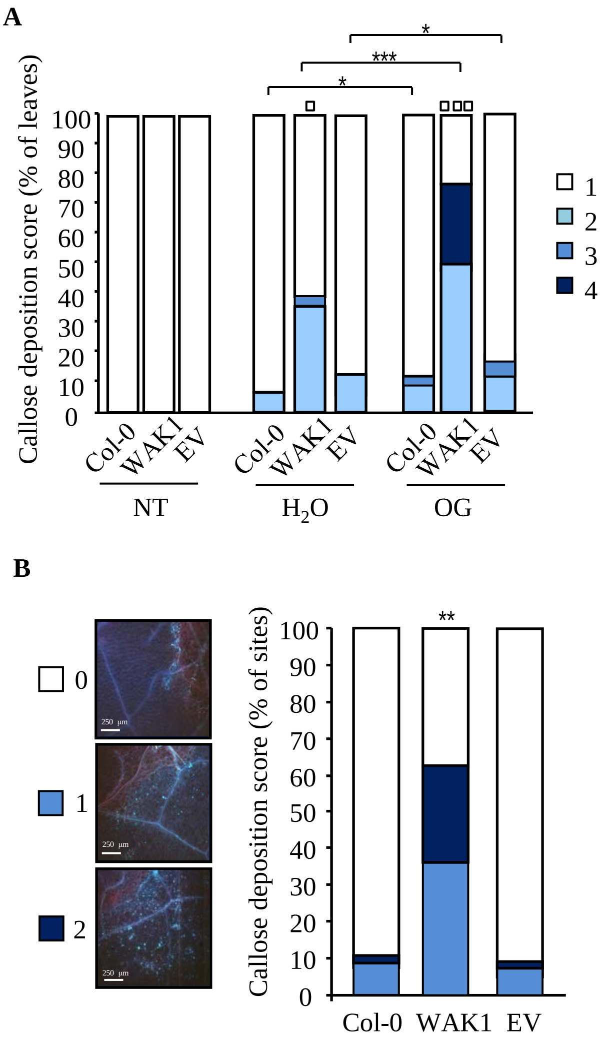

Supplementary Figure S6. A transgenic line transformed with the empty vector does not show enhanced OG-induced callose deposition and enhanced local response to wounding. (A) Leaves of Col-0, WAK1 or plants carrying the empty vector (EV) were sprayed with H_2O and OG (50 µg ml^{-1}) and stained after 24 h with aniline blue for callose visualization. Callose deposition is expressed as a score as indicated in Fig. 3A. The histograms show the percentage of leaves with a specific callose deposition score. White squares directly above bars indicate statistically significant difference between transgenic WAK1 and control plants, Col-0 and EV, within each treatment. Asterisks above connection lines indicate statistically significant difference between water and OG treatment within each genetic background, according to Fisher's exact test (* or white square $p < 0.05$; *** $p < 1x10^{-4}$). Two independent experiments were performed (n = 12 in each experiments). (B) Leaves were wounded by forceps and stained after 24 h with aniline blue for callose visualization. Callose intensity in a region surrounding the wound site (the proximal region) was evaluated according to a score scale that varies between 0 (no deposition), 1 (few deposits) and 2 (dense deposits). Representative callose deposition images for each score are shown on the left; all images are at the same scale. Bars, 250 µm. Histograms show the percentage of wound sites with a specific callose deposition score. Experiments were repeated two times (n = 12) with similar results. Asterisks indicate statistically significant difference between control and transgenic plants, according to Fisher's exact test (* $p < 1x10^{-3}$; ** $p < 1x10^{-4}$).

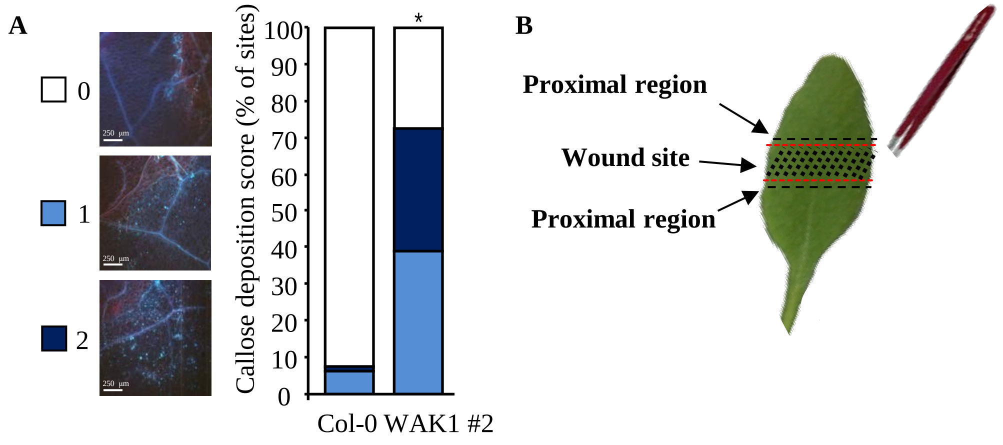

Supplementary Figure S7. WAK1 plants show enhanced local response to wounding. (A) A second transgenic line overexpressing WAK1 (line #2) shows enhanced local response to wounding. Leaves were wounded by forceps and stained after 24 h with aniline blue for callose visualization. Callose intensity in the wound proximal region was expressed by different score as indicated on the left; all images are at the same scale, bars 250 µm. The histograms on the right show the percentage of wound sites with a specific callose deposition score. Experiments were repeated five times (n = 12) with similar results. Asterisks indicate statistically significant difference between control and transgenic plants, according to Fisher's exact test (* $p < 1x10^{-4}$). (B) Schematic drawing showing a wounded leaf as used in our gene expression analysis and indicating the proximal region; the red dotted lines indicate the edge of the wound site.

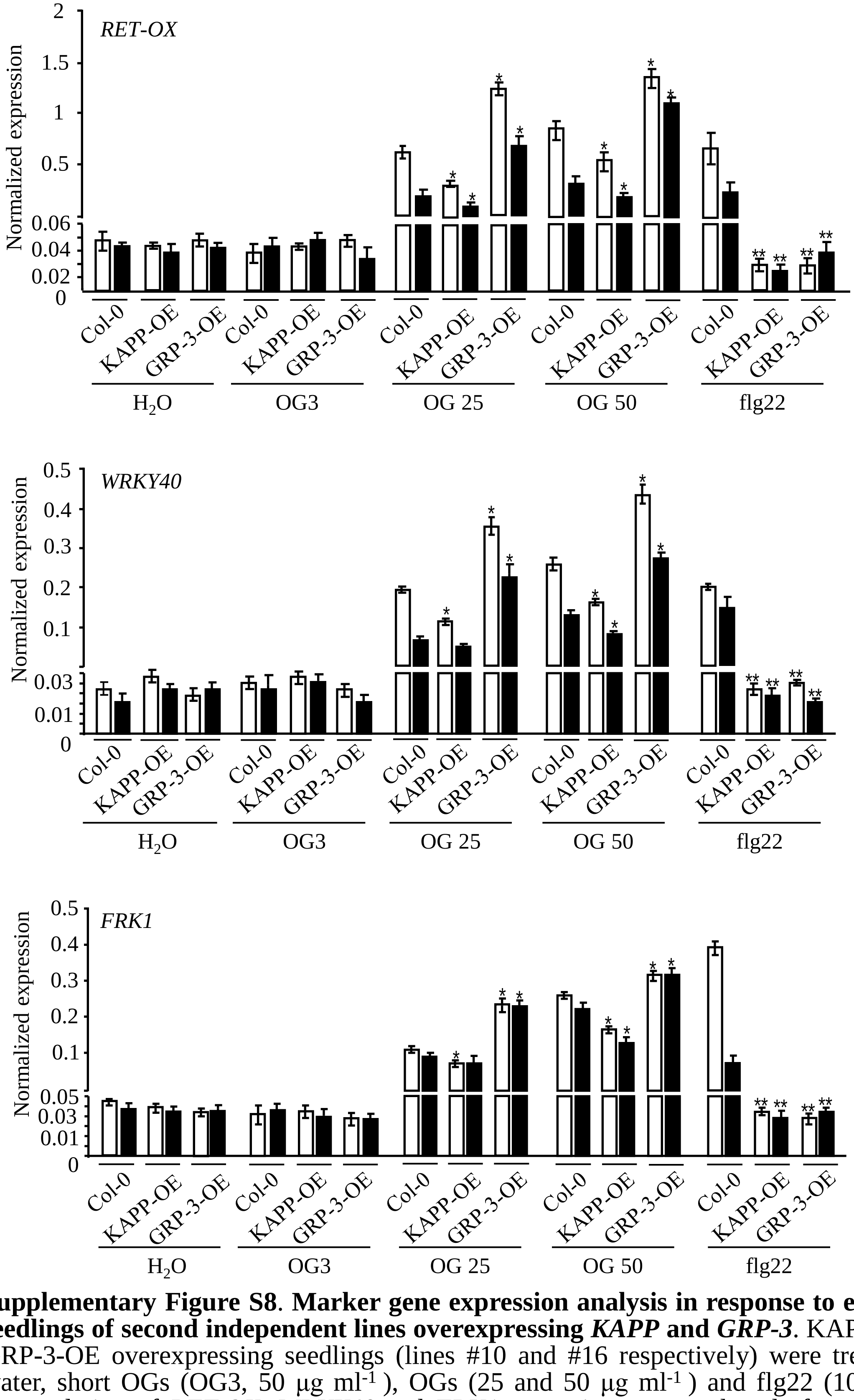

Supplementary Figure S8. **Marker gene expression analysis in response to elicitors in seedlings of second independent lines overexpressing *KAPP* and *GRP-3***. KAPP-OE and GRP-3-OE overexpressing seedlings (lines #10 and #16 respectively) were treated with water, short OGs (OG3, 50 µg ml^{-1}), OGs (25 and 50 µg ml^{-1}) and flg22 (10 nM) and accumulation of *RET-OX*, *WRKY40* and *FRK1* transcripts was analyzed after 1 h (white bar) and 3 h (black bar) by qRT-PCR, using *UBQ5* for normalization. Transcript levels are expressed as the gene/*UBQ5* ratio (normalized expression). Values are means (± SE) of two independent experiments (n = 20, in each experiment). Asterisks indicate statistically significant differences between elicitor treatment of overexpressing seedlings and Col-0, according to Student's t test (*, $p < 0.05$; **, $p < 1x10^{-3}$).

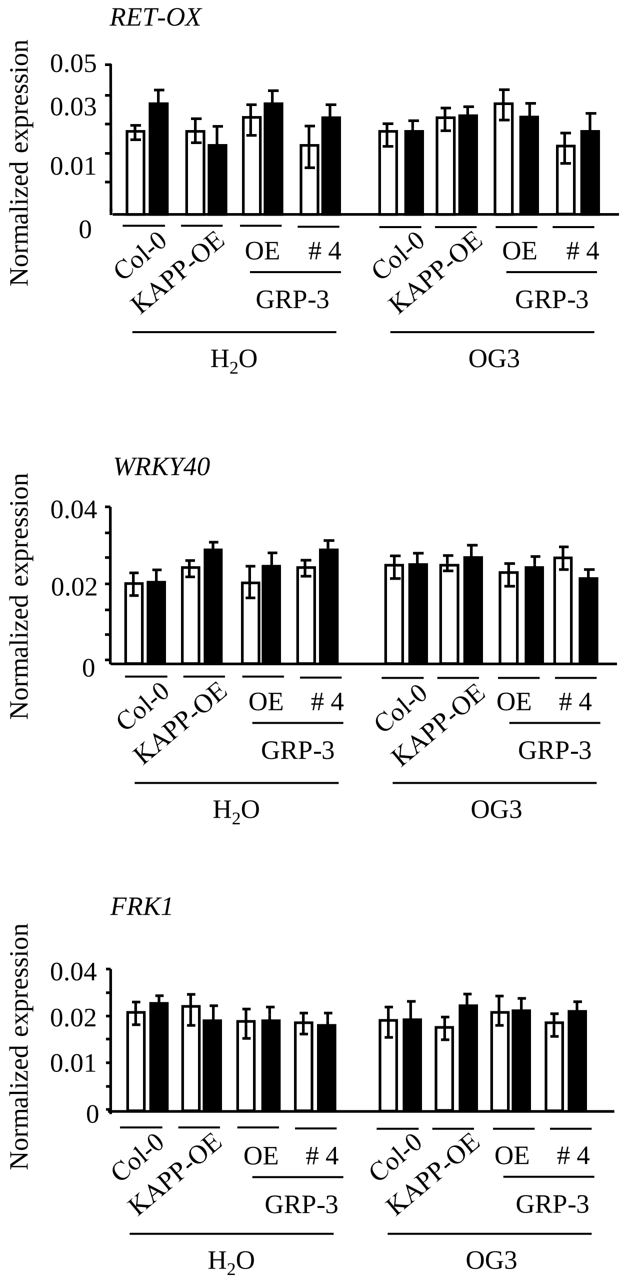

Supplementary Figure S9. Treatment with short OGs do not induce expression of defense response genes in Arabidopsis seedlings overexpressing *KAPP* and *GRP-3*. KAPP-OE (line #7), GRP-3-OE (line #17) and GRP-3 (line #4) seedlings were treated with water and short OGs (OG3, 50 µg ml^{-1}), and accumulation of *RET-OX*, *WRKY40* and *FRK1* transcripts was analyzed after 1 h (white bar) and 3 h (black bar) by qRT-PCR, using *UBQ5* for normalization. Transcript levels are expressed as the gene/*UBQ5* ratio (normalized expression). Values are means (± SE) of two independent experiments (n = 20, in each experiment).

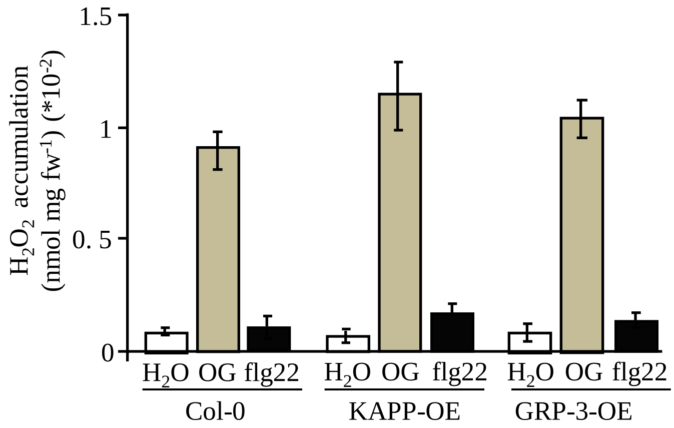

Supplementary Figure S10. Elicitor-induced production of extracellular hydrogen peroxide in KAPP and GRP-3 overexpressing seedlings. KAPP-OE (line #7) and GRP-3-OE (line #17) seedlings were treated with water (white bar), OGs (50 µg ml^{-1}, gray bar) and flg22 (100 nM, black bar) and accumulation of hydrogen peroxide in the culture medium was measured by a xylenol orange-based assay. Results are means of two independent experiments (± SE; n = 40 in each experiment).

Supplementary Table S1: Primers sequences used to characterization of mutant lines and to generate the constructs

Gene	AGI code	Forward Primer	Reverse Primer
KAPP (genotyping)	At5g19280	ATCATGATGCTTTTCTCGTGG	TCACTGGTTTGTCCTTGGATC
GRP-3 (genotyping)	At2g05520	ACATCATTAGCCAACGCTTTG	TTTCCTCCATTGTCACCGTAG
KAPP	At5g19280	ATGCAGAATTCATGGCGATGATAGGGATGAA	CCATCACAAACACAAAACAG
GRP-3	At2g05520	CCAGGAGGAGGTAATTACCA	TAGTGACCGGGCTGAGTC
WAK1	At1g21250	GGGGACAAGTTTGTACAAAAAAGCAGGCTCCATGAAGGTGCAGGAGGGTTT	GGGGACCACTTTGTACAAGAAAGCTGGGTAGCGGCCAGTTTCAATGTCCA
EFR	At5g20480	GATACCCCGGGATGAAGCTGTCCTTTTCACTT	GATACTTAATTAACTACATAGTATGCATGTCCG
GRP-3 (pDONR221/Zeo)	At2g05520	GGGGACAAGTTTGTACAAAAAAGCAGGCTCCATGGCTCCAAGGCTTTGGTT	GGGGACCACTTTGTACAAGAAAGCTGGGTAGTGACGGGCTGAGTCTGA
GRP-3 (pSAT6-RFP-N1)	At2g05520	ATGCAAAGCTTATGGCTTCCAAGGCTTTGG	TGCATCCTAGGGTGACCGGGCTGAGTCTGA
GRP-3 -RFP		GGGGACAAGTTTGTACAAAAAAGCAGGCTCCATGGCTTCCAAGGCTTTGGTT	GGGGACCACTTTGTACAAGAAAGCTGGGTATTAGGCGCCGGTGGA

Supplementary Table S2. Primer sequences used in gene expression analysis

**Gene**	**AGI code**	**Forward Primer**	**Reverse Primer**
UBQ5	At3g62250	GTTAAGCTCGCTGTTCTTCAGT	TCAAGCTTCAACTCCTTCTTTC
RET-OX	At1g26380	CGAACCCTAACAACAAAAAC	GACGACACGTAAGAAAGTCC
WRKY40	At1g80840	GATCCACCGACAAGTGCTTT	AGGGCTGATTTGATCCCTCT
FRK1	At2g19190	TGCACTTACCCTCCTTCG	GACAGTAGAAGCCGGTTGGT
RAP2	At1g78080	TTATTACCCGGATTCAACGTT	CCGTAAGCGAAACAAGATCC
WR3	At5g50200	GACCTGCCCACACAAGATCA	TGGAGGCAATATCTAGGGACGC
EFR	At5g20480	GGGTAATCTTAGGGCTGATT	CTGGACGAGT TATTTCCAAG
WAK1	At1g21250	ACAGCACTTGTCTCGATTCT	TCTTTACGCTTGCAGCTCAT
